# RARα2 and PML-RAR similarities in the control of basal and retinoic acid induced myeloid maturation of acute myeloid leukemia cells

**DOI:** 10.18632/oncotarget.10556

**Published:** 2016-07-13

**Authors:** Maurizio Gianni, Maddalena Fratelli, Marco Bolis, Mami Kurosaki, Adriana Zanetti, Gabriela Paroni, Alessandro Rambaldi, Gianmaria Borleri, Cecile Rochette-Egly, Mineko Terao, Enrico Garattini

**Affiliations:** ^1^ Laboratory of Molecular Biology, IRCCS-Istituto di Ricerche Farmacologiche “Mario Negri”, 20156 Milano, Italy; ^2^ Hematology and Bone Marrow Transplant Unit, Azienda Ospedaliera Papa Giovanni XXIII, 24127 Bergamo, Italy; ^3^ Department of Functional Genomics and Cancer, IGBMC (Institut de Génétique et de Biologie Moléculaire et Cellulaire), INSERM, U964, CNRS, UMR7104, Université de Strasbourg, 67404 Illkirch Cedex, France

**Keywords:** RARα2, retinoic acid, AML, silencing, PML-RAR

## Abstract

Treatment of acute promyelocytic leukemia (APL) with *all-trans* retinoic acid (ATRA) is the first example of targeted therapy. In fact, the oncogenic fusion-protein (PML-RAR) typical of this leukemia contains the retinoid-nuclear-receptor RARα. PML-RAR is responsible for the differentiation block of the leukemic blast. Besides PML-RAR, two endogenous RARα proteins are present in APL blasts, i.e. RARα1 and RARα2. We developed different cell populations characterized by PML-RAR, RARα2 and RARα1 knock-down in the APL-derived *NB4* cell-line. Unexpectedly, silencing of PML-RAR and RARα2 results in similar increases in the constitutive expression of several granulocytic differentiation markers. This is accompanied by enhanced expression of the same granulocytic markers upon exposure of the *NB4* blasts to ATRA. Silencing of PML-RAR and RARα2 causes also similar perturbations in the whole genome gene-expression profiles of vehicle and ATRA treated *NB4* cells. Unlike PML-RAR and RARα2, RARα1 knock-down blocks ATRA-dependent induction of several granulocytic differentiation markers. Many of the effects on myeloid differentiation are confirmed by over-expression of RARα2 in *NB4* cells. RARα2 action on myeloid differentiation does not require the presence of PML-RAR, as it is recapitulated also upon knock-down in PML-RAR-negative *HL-60* cells. Thus, relative to RARα1, PML-RAR and RARα2 exert opposite effects on APL-cell differentiation. These contrasting actions may be related to the fact that both PML-RAR and RARα2 interact with and inhibit the transcriptional activity of RARα1. The interaction surface is located in the carboxy-terminal domain containing the D/E/F regions and it is influenced by phosphorylation of Ser-369 of RARα1.

## INTRODUCTION

Acute-promyelocytic-leukemia (APL) is characterized by a t(15:17) chromosomal translocation involving *PML* and *RARA*, which results in the expression of the oncogenic PML-RAR fusion protein [1–3] and a block in the myeloid maturation pathway [4]. The cyto-differentiating agent *all-trans* retinoic acid (ATRA) is used in the treatment of APL and it has changed the natural history of the disease [5–9].

The biological action of ATRA is mediated by RAR and RXR nuclear receptors (*NRs*). RARα, RARβ, RARγ, RXRα, RXRβ and RXRγ are ligand-activated transcription-factors controlling the expression of target genes [10, 11]. The *NR* active forms consist of RAR/RXR heterodimers, in which the RAR moiety is responsible for ligand-binding [12–16]. ATRA binds/activates RARα, RARβ and RARγ with the same efficiency [17, 18]. The ligand-binding region of RARs is located in the carboxy-terminal E-domain, which is maintained in PML-RAR (Supplementary Figure S1).

The molecular mechanisms underlying the differentiation block afforded by PML-RAR in APL blasts and those responsible for ATRA therapeutic activity are incompletely defined. PML-RAR may arrest the myeloid maturation of APL blasts exerting a dominant-negative effect on RARα. Indeed, PML-RAR binds RAREs (Retinoic Acid Responsive Elements) of RARα target-genes [19]. Part of PML-RAR action may also involve RARα-independent mechanisms, as the fusion-protein binds to a larger set of DNA target-sequences than RARα [19]. The relative contribution of PML-RAR and RARα to the differentiation process ignited by ATRA in APL blasts is also largely unknown. ATRA-induced PML-RAR degradation may release RARα from the dominant-negative effect exerted by the fusion-protein, permitting its ligand-dependent activation [2, 20, 21]. The situation is further complicated by the presence of three different RARα isoforms (Supplementary Figure S1).

Using the *NB4* model of APL and silencing/over-expression approaches, we provide evidence that PML-RAR and the RARα splicing-variant, RARα2, inhibit basal and ATRA-dependent myeloid differentiation. In *NB4* cells, knock-down of the major RARα splicing variant, RARα1, exerts opposite effects relative to PML-RAR and RARα2. RARα2 action on myeloid differentiation is recapitulated in PML-RAR-negative and ATRA-sensitive *HL-60* cells. PML-RAR and RARα2 directly bind/inhibit RARα1 transcriptional activity, indicating functional antagonism.

## RESULTS

### RARα2 is expressed, transcriptionally activated and degraded by ATRA in the APL-derived NB4 cell line

**Figure 1:**
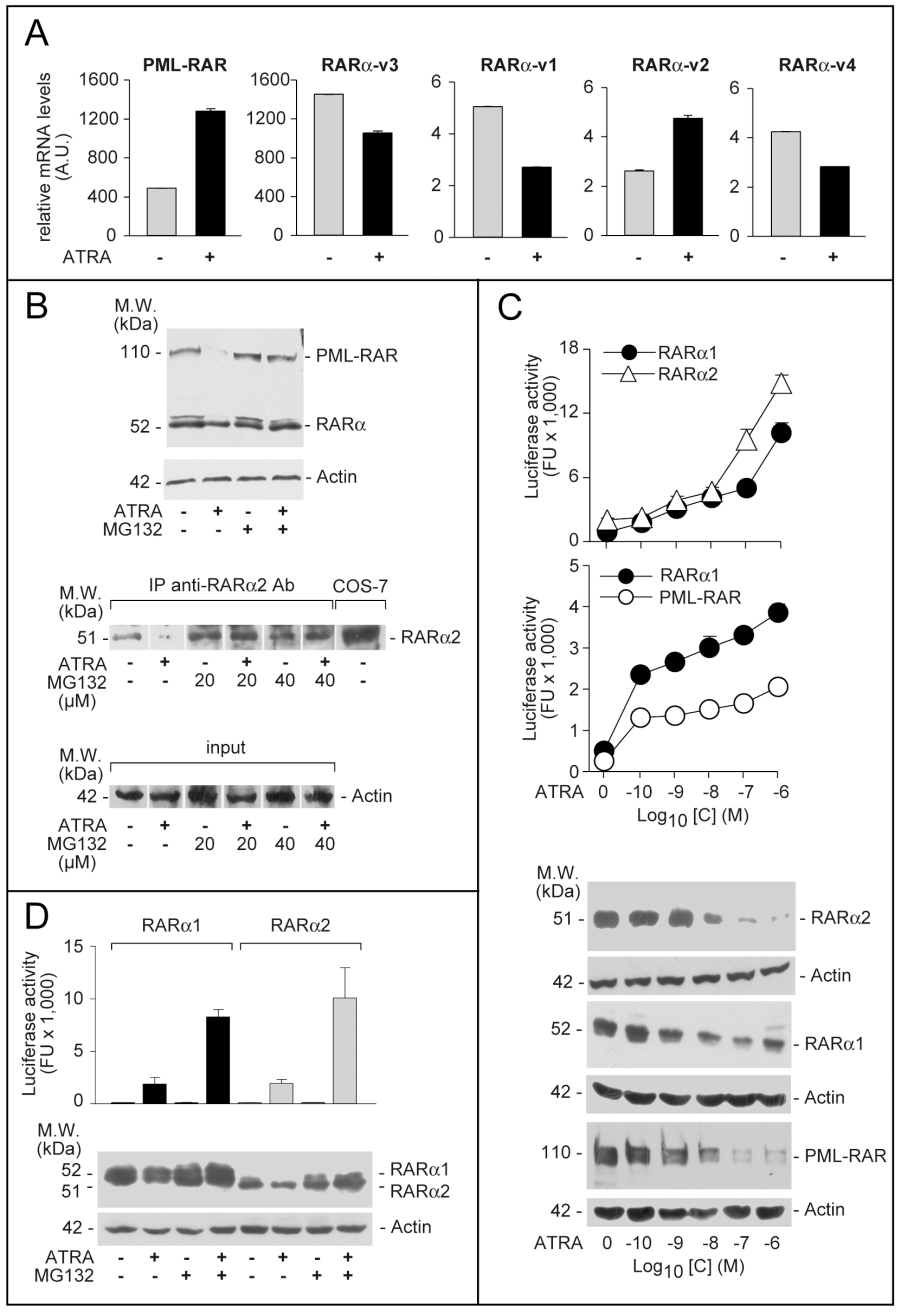
Expression, ATRA-dependent proteolytic degradation and transcriptional activity of PML-RAR, RARα2 and RARα1 **A**. *NB4* cells were treated with vehicle (DMSO) or ATRA (0.1 μM) for 48 hours. Total RNA was extracted and subjected to RT-PCR analysis using Taqman assays for the indicated mRNAs. The results are expressed as the mean±SD of 3 replicates. **B**. Upper: *NB4* cells were treated with vehicle (DMSO) or ATRA (0.1 μM) for 40 hours before addition of the proteasome inhibitor, MG132 (40 μM) for 8 hours. Total protein extracts were subjected to Western blot analysis with an anti-RARα antibody [RP alpha (F)]. Actin was used as a loading control. Lower: *NB4* cells were treated as above with vehicle (DMSO), ATRA (0.1 μM), the proteasome inhibitor, MG132 (20 and 40 μM) or ATRA+MG132. Cell extracts were immuno-precipitated with an anti-RARα2 antibody [Ab25alpha2(A2)] coupled to protein G-sepharose beads (IP = immuno-precipitation) and the immuno-precipitates were subjected to Western blot analysis with the same anti-RARα antibody used in the Upper panel. Equivalent amounts of protein extracts were used to immuno-precipitate RARα2, as indicated by the levels of actin in the extracts before addition of the anti-RARα2 antibody (input). *COS-7* = Total extracts of *COS-7* cells transfected with a *pcDNA3-RARα2* plasmid. The calculated molecular weight (M.W.) of the indicated proteins is shown on the left. **C**. *COS-7* cells were transfected with *pcDNA3-RARα2*, *pcDNA3-RARα1* and *pSG5-PML-RAR* plasmids and the retinoid dependent Luciferase reporter, *β2RARE-Luc*. Sixteen hours following transfection, cells were treated with DMSO or the indicated concentrations of ATRA for an extra 24 hours. Cell extracts were used for the measurement of luciferase activity and the indicated proteins by Western blot analysis. Luciferase activity data are expressed as the mean±SD of two replicates. **D**. *COS-7* cells were transfected as in (**C**). Sixteen hours following transfection, cells were treated with vehicle (DMSO) or ATRA (1 μM ) for 16 hours and vehicle or MG132 (40 μM) for an extra 8 hours. Cell extracts were used for the measurement of luciferase activity and the indicated proteins by Western blot analysis. Luciferase activity data are expressed as the mean±SD of two replicates.

Four RARα splicing-variant mRNAs, RARα-v1, RARα-v2, RARα-v3 and RARα-v4, are known (Supplementary Figure S1). RARα-v1 and RARα-v3 code for an identical protein (RARα1). RARα-v4 is translated into RARα4 lacking the DNA-binding *C*-region. RARα2, the RARα-v2 product, is devoid of the *A*-region [22]. We determined the levels of PML-RAR and RARα splicing-variants in *NB4* cells grown with and without ATRA (Figure 1A). In the absence of ATRA, large amounts of PML-RAR mRNA are measurable, while RARAα-v3 is the major endogenous RARα transcript, followed by RARα-v1, RARα-v2 and RARα-v4. PML-RAR and RARα-v2 mRNAs are induced by ATRA.

**Figure 2:**
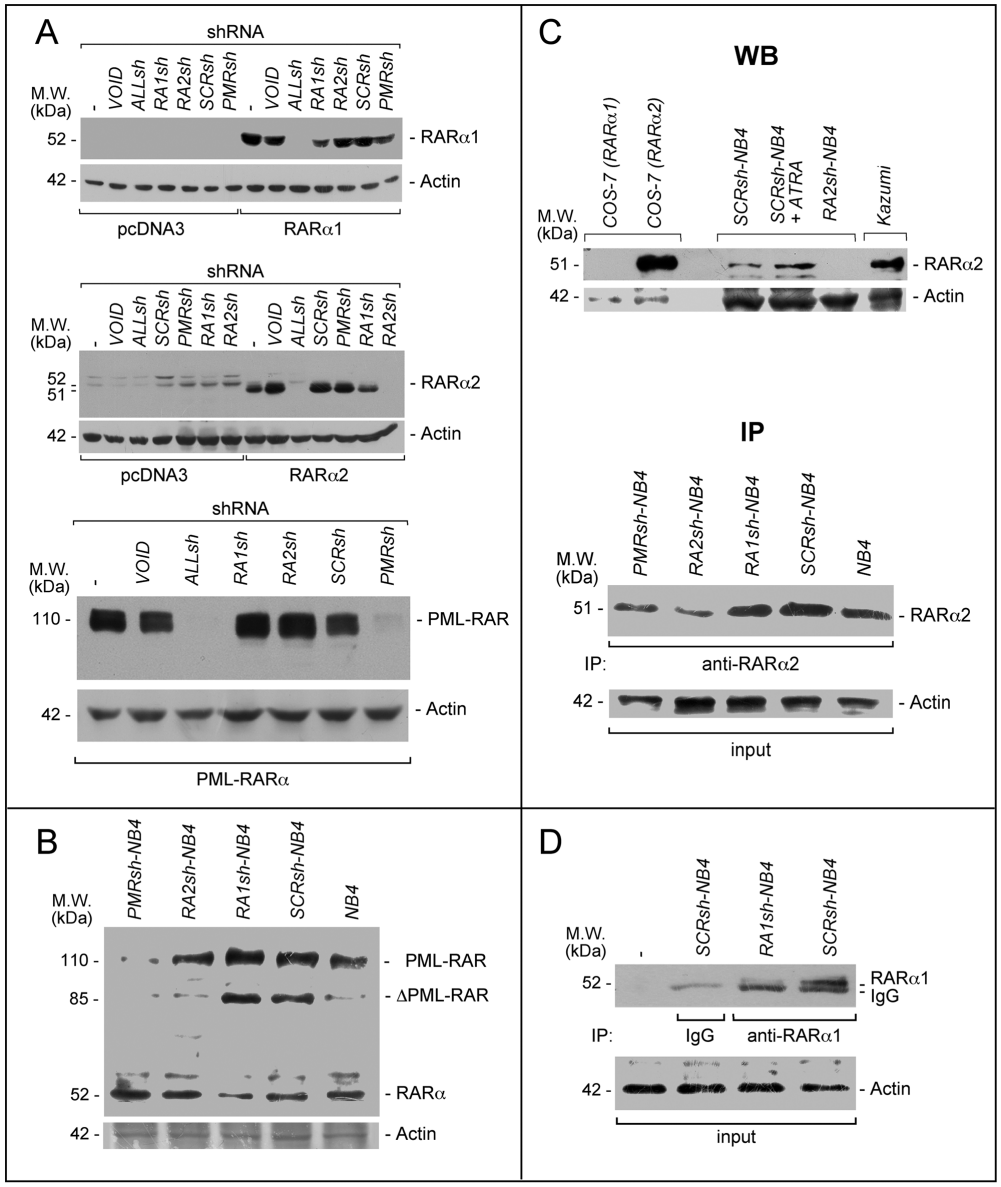
PML-RAR, RARα2 and RARα1 knock-down in COS-7 and NB4 cells **A**. *COS-7* cells were transiently transfected with the *pcDNA3, pcDNA3-RARα2*, *pcDNA3-RARα1* and *pSG5-PML-RAR* plasmids in the presence of the indicated shRNA-containing retroviral vectors and corresponding void vector (*VOID*). Sixteen hours following transfection, cell extracts were subjected to Western blot analysis using an anti-RARα antibody [RP alpha (F)]. Actin is used as a loading control. *ALLsh* = shRNA targeting RARα1 (RARα.v1 and RARα.v3 mRNAs), RARα2 (RARα.v2 mRNA) and RARα4 (RARα.v4 mRNA); *RA1sh* = shRNA targeting RARα1; *RA2sh* = shRNA targeting RARα2; *PMRsh* = shRNA targeting PML-RAR; *SCRsh* = scramble shRNA (negative control). The (-) symbol represents extracts from *COS-7* cell transfected in the absence of any shRNA. **B**. The indicated *NB4* cell populations stably infected with shRNAs targeting PML-RAR (*PMRsh-NB4*), RARα1 (*RA1sh-NB4*), RARα2 (*RA2sh-NB4*) or scramble shRNA (*SCRsh-NB4*) as well parental *NB4* cells (*NB4*) were grown under standard conditions for 48 hours. Cell extracts were subjected to Western blot analysis using the same anti-RARα antibody as in (**A**). Actin is used as a loading control. **C**. Upper (WB = Western Blots): Extracts from the indicated *COS-7* cells transfected with RARα1 and RARα2 expressing plasmids as well as the indicated *NB4* cells [see (B)], were subjected to Western blot analysis with anti-RARα2 [Ab25alpha2(A2)] and β-actin (loading control) antibodies. *SCRsh-NB4* = cell treated with vehicle (DMSO) for 24 hours; *SCRsh-NB4+ATRA* = cell treated with ATRA (1 μM) for 24 hours. *Kazumi* cells extracts are used as a control for RARα2 expression, as they contain high levels of the protein. Lower (IP = immunoprecipitations): Extracts from the indicated *NB4* cell populations and parental *NB4* cells [see (B)], were immuno-precipitated with an anti-RARα2 antibody [Ab25alpha2(A2)] coupled to Protein G sepharose beads. The immuno-precipitates were subjected to Western blot analysis with a different anti-RARα antibody [RP alpha (F)]. Equivalent amounts of protein extracts were used to immuno-precipitate RARα2, as indicated by the levels of actin in the extracts before addition of the anti-RARα2 antibody (input). **D**. Extracts from the indicated *NB4* cell populations [see (B)] were immuno-precipitated with an anti-RARα1 antibody [Ab10alpha1(A1)] or mouse immunoglobulin G (IgG) coupled to Protein G Sepharose beads or Protein G Sepharose beads alone (-). The immuno-precipitates were subjected to Western blot analysis with a different anti-RARα antibody [RP alpha (F)]. The actin loading control of the immuno-precipitation experiment is shown (input). The calculated molecular weight (M.W.) of each protein is indicated on the left of each blot.

High levels of PML-RAR and RARα proteins are highlighted by an antibody [RP-alpha-(F)] recognizing the *F*-region of the two receptors (Figure 1B). Although this antibody recognizes both RARα1 and RARα2, the RARα bands determined upon Western blot analysis (WB) are likely to correspond to RARα1. Indeed, when a selective anti-RARα2 antibody [Ab25alpha(A2)] is used, a specific RARα2 band is measurable only following immuno-precipitation (Figure 1B) or WB analysis with high amounts of extracts (see Figure 2C, upper). ATRA-treated *NB4* cells show the expected degradation of PML-RAR and RARAα1 proteins, which is associated with receptor activation [23, 24] and suppressed by the proteasome-inhibitor, MG132 (Figure 1B). Despite induction of the corresponding mRNA, the RARα2 protein band disappears upon ATRA challenge (Figure 1B). This effect is also consequent to proteasome-dependent RARα2 degradation, since MG132 blocks it (Figure 1B). ATRA causes a concentration-dependent stimulation of RARα1, RARα2 and PML-RAR transcriptional activity as well as a decrease in the relative protein levels in *COS-7* cells co-transfected with the *NRs* and a retinoid luciferase-reporter (Figure 1C). The ATRA-dependent decrease in RARα1, RARα2, and PML-RAR proteins is suppressed by MG132 (Figure 1D). As observed for RARα1 (Figure 1D) and PML-RAR (data not shown) [24, 25], inhibition of RARα2 degradation by MG132 increases the induction of luciferase activity by ATRA.

### Generation of NB4 derived cell populations stably and selectively silenced for PML-RAR, RARAα1 and RARα2

We designed shRNAs targeting the fusion site of PML-RAR as well as the *A*/*B*-regions of RARα1 and RARα2 (Supplementary Figure S2). The RARα1-targeting shRNAs recognize also RARα4 and are expected to silence it too. However, RARα4 is undetectable in *NB4* cells (data not shown) and it was not further considered. Following a preliminary screening based on transient transfection of *COS-7* cells with PML-RAR, RARAα1 or RARα2 cDNAs, we selected one shRNA for each receptor and one shRNA recognizing all the receptors (*ALLsh*). *ALLsh* efficiently down-regulates RARα1, RARα2 and PML-RAR in *COS-7* cells (Figure 2A). The RARα1, RARα2 and PML-RAR shRNAs (*RA1sh*, *RA2sh* and *PMRsh*) reduce the levels of the targets specifically, while the negative-control shRNA (*SCRsh*) retroviral construct and the void vector (*VOID*) exert no effect.

We isolated *NB4* cell populations with stable integration of the selected negative-control (*SCRsh-NB4*), the RARα1 (*RA1sh-NB4*), the RARα2 (*RA2sh-NB4*) and the PML-RAR (*PMRsh-NB4*) shRNAs. Specific knock-down of the targets is confirmed for *RA1sh-NB4* and *PMRsh-NB4* cells (Figure 2B), while the parental and *SCRsh-NB4* counterparts express similar levels of RARα1 and PML-RAR. The same amounts of PML proteins are evident in parental and *PMRsh-NB4* cells supporting the specificity of the PML-RAR targeting shRNA (Supplementary Figure S3). Following WB (Figure 2C-upper) or immuno-precipitation (Figure 2C-lower) with the anti-RARα2 antibody [Ab25a(A2)], a remarkable and selective down-regulation of RARα2 is observed in *RA2sh-NB4* cells. If similar immuno-precipitation experiments are performed with an anti-RARα1 antibody, a decrease in the amounts of RARα1 is evident in *RA1sh-NB4* relative to *SCRsh-NB4* cells, confirming the WB results obtained on cellular extracts (Figure 2D).

### PML-RAR and RARα2 silencing increases differentiation of NB4 cells in the absence or presence of ATRA, while RARα1 silencing exerts opposite effects in the presence of the retinoid

**Figure 3:**
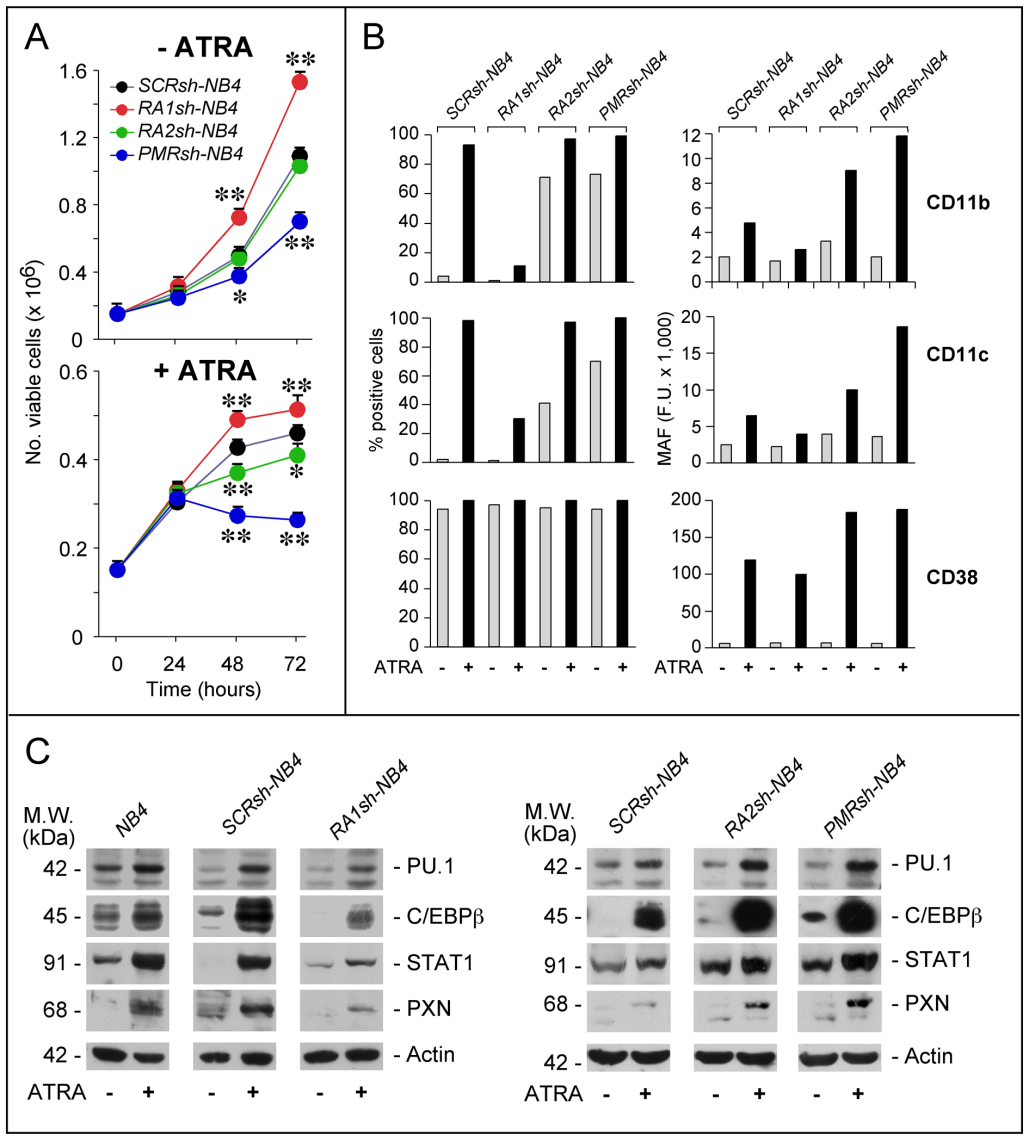
Effects of PML-RAR, RARα2 and RARα1 knock-down on the growth and differentiation of NB4 cells **A**. The indicated *NB4* cell populations stably infected with shRNAs targeting PML-RAR (*PMRsh-NB4*), RARα1 (*RA1sh-NB4*), RARα2 (*RA2sh-NB4*) or the control scramble shRNA (*SCRsh-NB4*) were grown in the presence of vehicle (DMSO) or ATRA (1 μM) for the indicated amount of time. The number of viable cells determined after staining with trypan blue is indicated. Each point is the mean±S.D. of three replicate cultures. ** = Significantly different relative to the corresponding *SCRsh-NB4* time point (p<0.01 after Student's t-test); * = Significantly different relative to the corresponding *SCRsh-NB4* time point (p<0.05 after Student's t-test). **B**. The indicated *NB4* cell populations [see (A)] were grown in the presence of vehicle (DMSO) or ATRA (1 μM) for 72 hours. Cells were subjected to FACS analysis for the indicated markers. The column graphs on the left indicate the percentage of CD11b-, CD11c- and CD38-positive cells. The graphs on the right indicate the MAF (mean-associated-fluorescence) values determined. The results are representative of two independent experiments. **C**. The indicated *NB4* cell populations were treated as in (B) for 48 hours. Cell extracts were subjected to Western blot analysis for the indicated proteins. Actin is used as a loading control. The results shown in the upper and lower panels were obtained in separate experiments. Each line shows cropped lanes of the same gel, hence the results can be compared across the lanes, as they were obtained with the same exposure time. The calculated molecular weight (M.W.) of each protein is indicated on the left. The results are representative of at least two independent experiments.

The consequences of RARα1, RARα2 and PML-RAR knock-down on *NB4* growth were evaluated in the absence/presence of ATRA (Figure 3A). In the absence of ATRA, *RA1sh-NB4* and *PMRsh-NB4* cells grow faster and more slowly, respectively, than *SCRsh-NB4* blasts. No difference is observed between *RA2sh-NB4* and *SCRsh-NB4* cells. Both PML-RAR and RARα2 silencing enhances ATRA growth-inhibitory action, while RARα1 knock-down exerts opposite effects.

Under basal conditions, a significant fraction of *PMRsh-NB4* and *RA2sh-NB4* cells show morphological signs of granulocytic maturation which are not observed in *RA1sh-NB4* cells, such as nuclear lobulation, increased cytoplasmic/nuclear ratio and appearance of cytoplasmic granules/vesicles (Supplementary Figure S4). As expected, ATRA induces morphological features of granulocytic differentiation in the majority of *SCRsh-NB4* cells. While similar features are observed in ATRA-treated *PRsh-NB4* and *RA2sh-NB4* cells, differentiation is much less evident in *RA1sh-NB4* cells. In the absence of ATRA, *SCRsh-NB4* blasts show the same low levels of CD11b and CD11c myeloid differentiation markers [26] (Figure 3B). Exposure to ATRA renders *SCRsh-NB4* cells highly positive for the two markers and it increases CD11b as well as CD11c mean-associated-fluorescence (MAF). In *RA1sh-NB4* cells, ATRA-dependent induction of these markers is suppressed, as indicated by CD11b-/CD11c-positivity and MAF. Relative to *SCRsh-NB4* cells, a substantial increase in CD11b-/CD11c-positivity is already observed in *PMRsh-NB4* and *RA2sh-NB4* blasts grown under basal conditions. The phenomenon is accompanied by enhanced MAF values after exposure to ATRA. Thus, *RA2sh-NB4* and *PMRsh-NB4* cells show similar patterns of basal and ATRA-dependent CD11b/CD11c expression. We also defined the action of RARAα1, RARα2 and PML-RAR shRNAs on 3 transcription factors controlling APL blast granulocytic maturation, *i.e*. PU.1 [27, 28], cEBPβ [29, 30] and STAT1α [31] (Figure 3C). RARAα1 knock-down reduces the induction of PU.1, cEBPβ and STAT1α observed in ATRA-exposed parental and *SCRsh-NB4* blasts. In contrast, *PMRsh-NB4* and *RA2sh-NB4* cells show higher basal levels of STAT1α and cEBPβ as well as enhanced induction of PU.1, STAT1α and cEBPβ by ATRA.

To evaluate the effects of PML-RAR, RARα2 and RARAα1 silencing on direct retinoid-targets, we focused on CD38 (Figure 3B) and paxillin (PXN) (Figure 3C) which are up-regulated by ATRA in *NB4* and other cell types [32–35]. Over 90% of all shRNA-infected cells are CD38^+^. ATRA increases CD38-MAF in *SCRsh-NB4* and *RA1sh-NB4* cells. The ATRA-dependent effect is considerably enhanced in *PMRsh-NB4* and *RA2sh-NB4* blasts. This enhancement is not evident in *RA1sh-NB4* cells, which show a slight reduction of the ATRA-dependent CD38-MAF increase in *SCRsh-NB4* blasts. Similar expression patterns are observed in the case of PXN. Thus, RARα2 and PML-RAR are negative determinants of myeloid differentiation and they down-regulate direct retinoid-responsive genes.

To evaluate whether the negative action of RARα2 on myeloid differentiation is specific to APL and dependent on PML-RAR expression, we performed studies in the *PML-RAR^−^* and ATRA-sensitive *HL-60* model [45, 46]. In *HL-60* cells, RARα-v3 is most abundant followed by RARα-v1 and RARα-v2 mRNAs, while negligible amounts of RARα-v4 are measured (Supplementary Figure S5A). ATRA up-regulates RARα-v2 and RARα-v3 mRNAs. Relative to control *HL-60* populations (*SCRsh-HL60* and *Void-HL60* cells), RARα2 knock-down (*RA2sh-HL60*) (Supplementary Figure S5B) reduces basal cell-growth and enhances ATRA anti-proliferative action (Supplementary Figure S5C). In basal conditions, RARα2 knock-down increases the number of CD11b^+^ *RA2sh-HL60* cells (Supplementary Figure S5D). Following ATRA exposure (0.1 and 1.0 μM), the number of CD11b^+^ cells and CD11b-MAF values are higher in *RA2sh-HL60* than *SCRsh-HL60* or *Void-HL60* cells. Both ATRA concentrations enhance CD11c-MAF induction in *RA2sh-HL60* relative to control cells. Finally, ATRA-dependent up-regulation of STAT1α, cEBPβ and PU.1 are enhanced by RARα2 knock-down (Supplementary Figure S5E). As for the consequences of RARα2 knock-down on the direct retinoid-responsive genes, all *RA2sh-HL60* cells are CD38^+^, while *SCRsh-HL60* and *Void-HL60* blasts are CD38^−^ (Supplementary Figure S5F). As expected, ATRA renders *SCRsh-HL60* and *Void-HL60* cells CD38^+^ and increases CD38-MAF in *SCRsh-HL60*, *Void-HL60* and *RA2sh-HL60* to the same extent. RARα2 knock-down stimulates ATRA-dependent induction of paxillin (Supplementary Figure S5E) as well as c-EBPε and CYP-26A1 mRNAs (Supplementary Figure S5G). All this confirms and extends the *NB4* data, indicating that RARα2 exerts a negative action on myeloid maturation independently of PML-RAR expression.

### RARα2 over-expression reduces ATRA-dependent differentiation of NB4 cells

**Figure 4:**
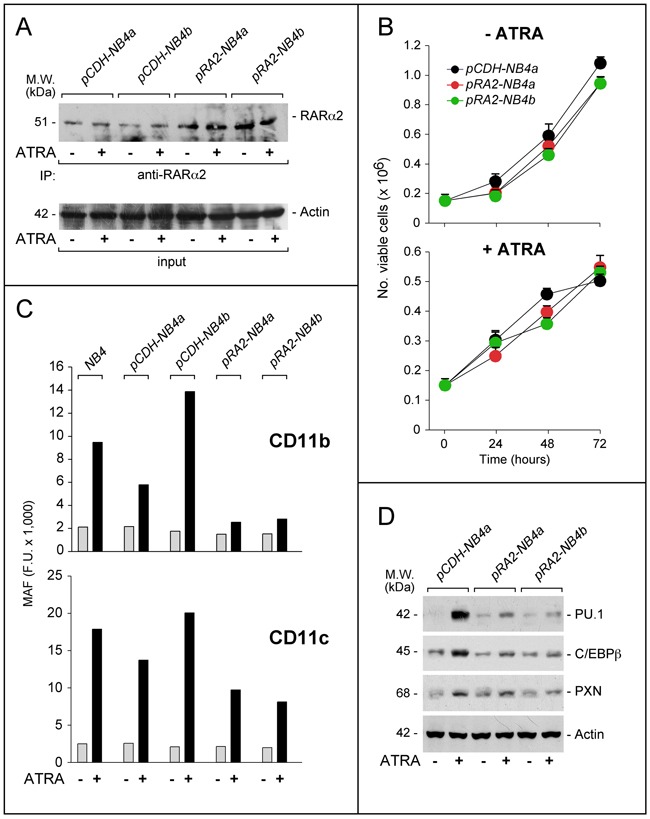
Effects of RARα2 over-expression on the growth and differentiation of NB4 cells *NB4* cells were transfected with the *pCDH-RA2* plasmid and the corresponding void vector, *pCDH*. Two distinct RARα2 expressing (*pRA2-NB4a* and *pRA2-NB4b*) and two (*pCDH-NB4a* and *pCDH-NB4b*) cell populations were isolated. **A**. Cells were treated with ATRA (1 μM) for 24 hours. Cell extracts were immuno-precipitated with an anti-RARα2 antibody [Ab25alpha2(A2)] coupled to Protein G sepharose beads. The immuno-precipitates were subjected to Western blot analysis with a different anti-RARα antibody [RP alpha (F)]. Equivalent amounts of protein extracts were used to immuno-precipitate RARα2, as indicated by the levels of actin present in the extracts before addition of the anti-RARα2 antibody (input). **B**. Cells were treated with vehicle (DMSO) or ATRA (1 μM) for the indicated amount of time. The number of viable cells determined after staining with trypan blue is indicated. Each point is the mean±S.D. of three replicate cultures. **C**. Cells were grown in the presence of vehicle (DMSO) or ATRA (1 μM) for 72 hours and subjected to FACS analysis for the determination of CD11b and CD11c. The column graphs indicate the MAF (mean-associated-fluorescence) values determined. **D**. Cells were grown as in (C) and treated with vehicle (DMSO) or ATRA (1 μM) for 48 hours. Cell extracts were subjected to Western blot analysis for the indicated proteins. Actin is used as a loading control. The calculated molecular weight (M.W.) of each protein is indicated on the left.

To support the unexpected data obtained following RARα2 silencing in *NB4* cells, we took a specular approach and stably over-expressed the retinoid receptor in the same cellular context. To this purpose, we produced *NB4* cell populations stably transfected with a RARα2 plasmid (*pCDH-RA2*) or the void vector (*pCDH*) (Figure 4A). Independent control (*pCDH-NB4a; pCDH-NB4b*) and RARα2 over-expressing (*pRA2-NB4a*; *pRA2-NB4b*) cell populations were compared for their growth in the absence/presence of ATRA. In *pCDH-NB4a*, *pRA2-NB4a* and *pRA2-NB4b*, the basal growth and the anti-proliferative action of ATRA are similar (Figure 4B). In untreated parental, *pCDH-NB4* and *pRA2-NB4* cells, the same low levels of CD11b- and CD11c-MAF are observed (Figure 4C). However, the ATRA-dependent increase of CD11b-MAF in parental, *pCDH-NB4a* and *pCDH-NB4b* cells is suppressed in *pRA2-NB4a* and *pRA2-NB4b* cells. RARα2 over-expressing blasts show also a substantial inhibition of ATRA-dependent CD11c induction. In vehicle and ATRA-treated *pRA2-NB4a*, *pRA2-NB4b* and *pCDH-NB4a* cells, a similar trend is evident, if we compare the levels of PU.1, cEBPβ and paxillin, (Figure 4D). Hence, RARα2 over-expression and RARα2 knock-down exert opposite effects on the myeloid-associated markers considered.

### Silencing of PML-RAR and RARα2 exerts similar effects on the NB4 whole-genome gene-expression profiles

**Figure 5:**
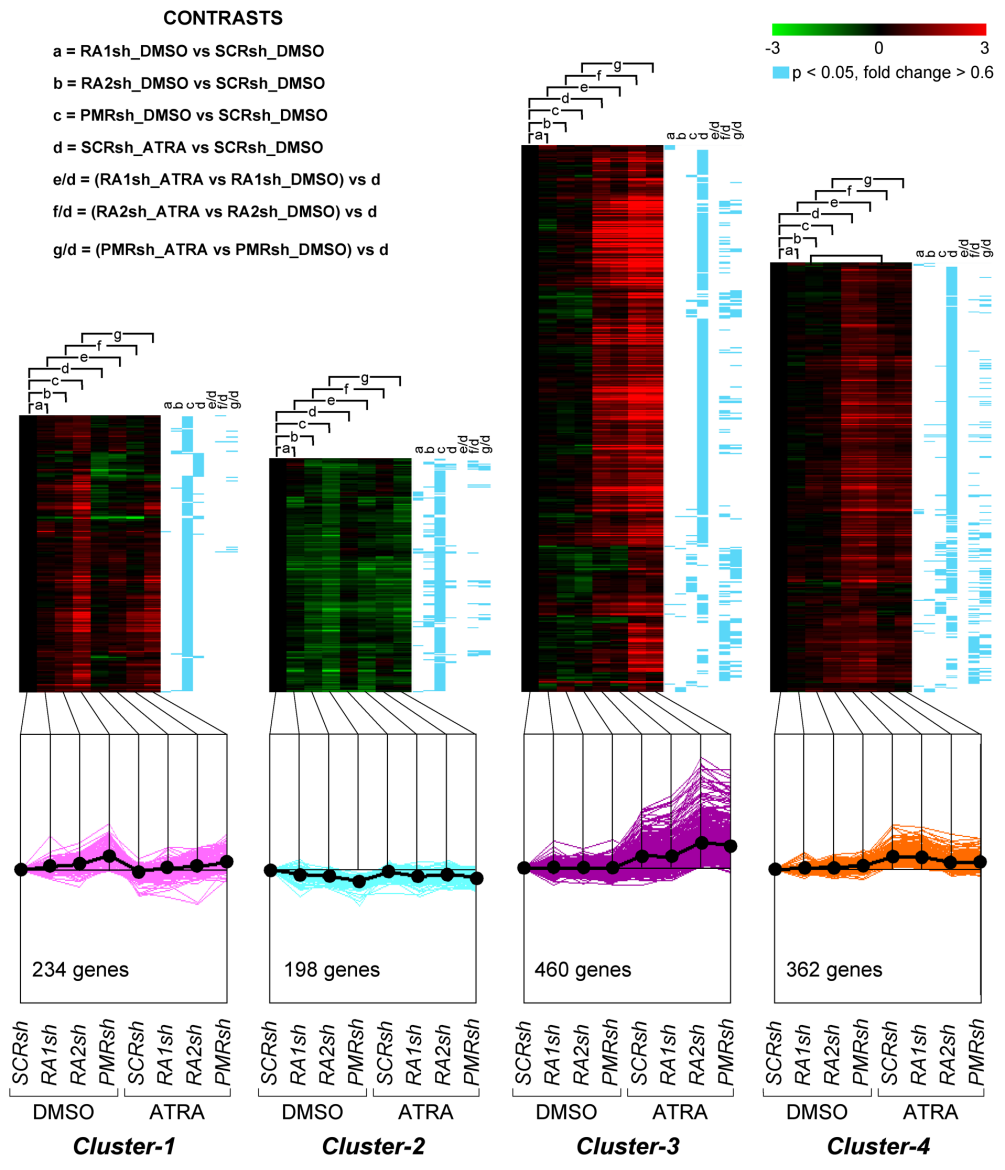
Perturbations of the basal and ATRA-dependent gene expression caused by PML-RAR, RARα2 and RARα1 knock-down in NB4 cells Classification by K-means clustering: Cluster-1 through Cluster-4 genes. The indicated *NB4* cell populations stably infected with PML-RAR (*PMRsh-NB4*), RARα1 (*RA1sh-NB4*), RARα2 (*RA2sh-NB4*) and scramble (*SCRsh-NB4*) shRNAs were grown in the presence of vehicle (DMSO) or ATRA (0.1 μM) for 48 hours. Total RNA was used to perform whole-genome gene-expression microarray experiments. For each experimental group, data are reported as the log_2_ of the ratio *vs* basal expression in *SCRsh-NB4* cells (SCRsh_DMSO). The genes regulated in at least one of the experimental conditions are grouped into eight Clusters and the figure illustrates *Cluster-1* to *Cluster-4*. The upper graphs are heat-maps generated by hierarchical clustering using Pearson's distances. The statistical significance of the indicated comparisons (CONTRASTS, p<0.05, log_2_ ratio > 0.6 or < -0.6) is shown on the right by the blue lines. The lower line graphs show the global expression profiles.

**Figure 6:**
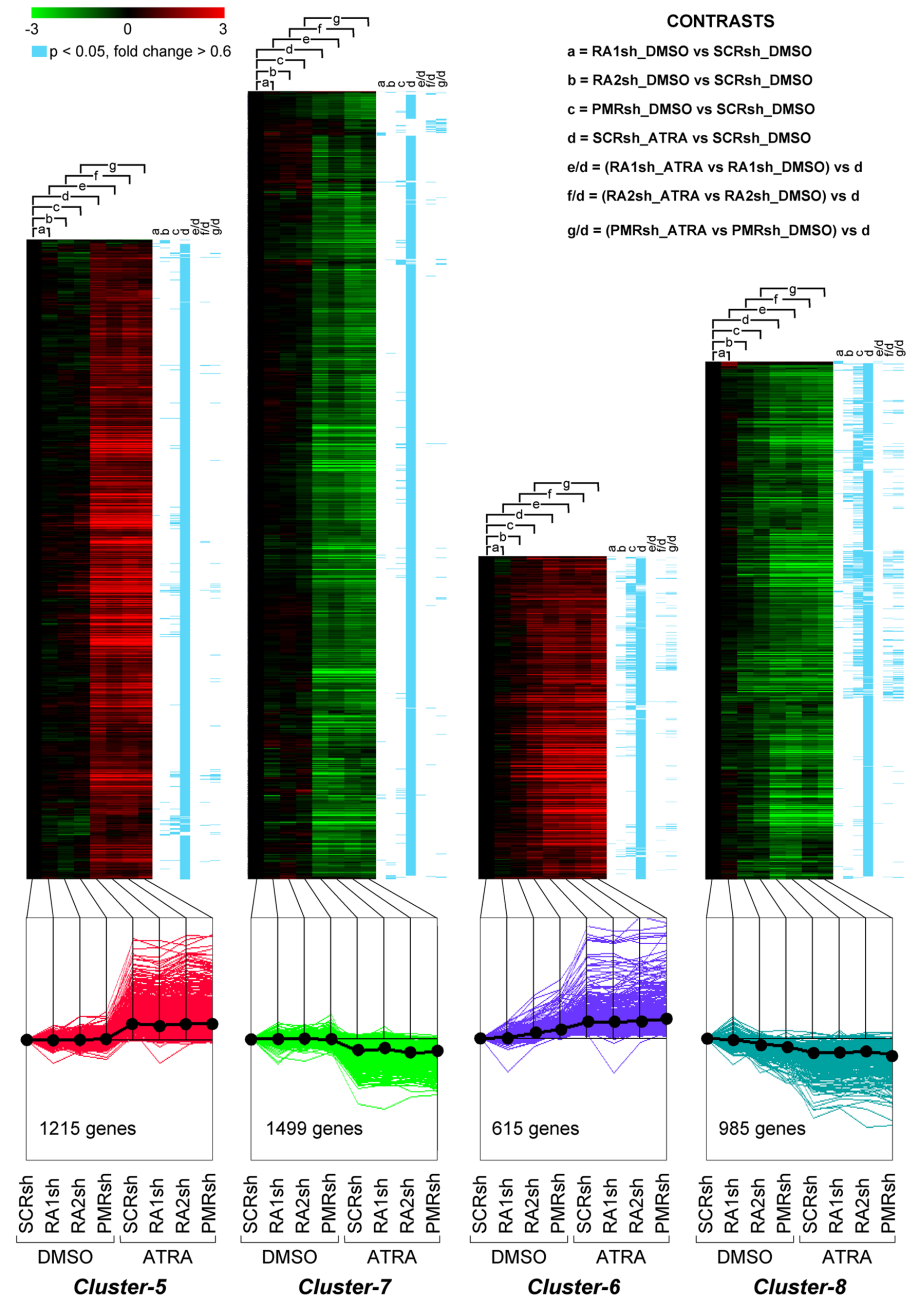
Perturbations of the basal and ATRA-dependent gene expression caused by PML-RAR, RARα2 and RARα1 knock-down in NB4 cells Classification by K-means clustering: Cluster-5 through Cluster-8 genes. The indicated *NB4* cell populations stably infected with PML-RAR (*PMRsh-NB4*), RARα1 (*RA1sh-NB4*), RARα2 (*RA2sh-NB4*) and scramble (*SCRsh-NB4*) shRNAs were grown in the presence of vehicle (DMSO) or ATRA (0.1 μM) for 48 hours. Total RNA was used to perform whole-genome gene-expression microarray experiments. For each experimental group, data are reported as the log_2_ of the ratio *vs* basal expression in *SCRsh-NB4* cells (SCRsh_DMSO). The genes regulated in at least one of the experimental conditions are grouped into eight Clusters and the figure illustrates *Cluster-5* to *Cluster-8*. The upper graphs are heat-maps generated by hierarchical clustering using Pearson's distances. The statistical significance of the indicated comparisons (CONTRASTS, p<0.05, log_2_ ratio > 0.6 or < -0.6) is shown on the right by the blue lines. The lower line graphs show the global expression profiles.

We compared the gene-expression profiles of *PMRsh-NB4*, *RA2sh-NB4*, *RA1sh-NB4* and *SCRsh-NB4* cells exposed to vehicle or ATRA for 48 hours [36, 37]. The time point was selected because it precedes terminal differentiation of *NB4* cells and it is characterized by large and ATRA-dependent variations of the whole-genome gene-expression profiles in parental *NB4* cells. We identified 5,567 genes whose expression is significantly modified in at least one of the comparisons considered (p<0.05, BH, 0.6 fold-change threshold). Differentially expressed genes can be classified into 8 clusters according to their expression pattern by the K-means algorithm (Supplementary Table S1). *Cluster-1* and *Cluster-2* genes are up- or down-regulated in *PMRsh-NB4* cells under basal conditions and ATRA exerts no or very limited effects on their expression (Figure 5). In *PMRsh-NB4* cells, up-/down-regulation of the majority of genes reaches statistical significance. Although changes are smaller and often lack significance, the same regulation pattern of *Clusters-1*/*-2* genes is observed in *RA2sh-NB4* cells. *Cluster-3*/*-4* genes are up-regulated by ATRA in *SCRsh-NB4* and *RA1sh-NB4* cells to the same extent. ATRA-dependent up-regulation of *Cluster-3* genes is enhanced in *PMRsh-NB4* and *RA2sh-NB4* cells, while down-regulation of *Cluster-4* genes is repressed following PML-RAR and RARα2 knock-down (Figure 5). The differences reach statistical significance in a considerable fraction of genes. Once again, PML-RAR and RARα2 act on common gene-sets which are regulated by these *NRs* in the same direction. *Clusters-5*/*-7* consist of numerous genes whose basal or ATRA-dependent expression is left generally unaffected by PML-RAR, RARα1 or RARα2 knock-down (Figure 6). *Cluster-5* genes are up-regulated, while *Cluster-7* genes are down-regulated by ATRA. Following ATRA treatment, *Clusters-6/-8* have similar profiles of expression relative to *Clusters-5/-7*. However, *Clusters-6/-8* genes are up-/down-regulated by PML-RAR and RARα2 knock-down also in basal conditions. Overall, the effects of RARα1 knock-down are small with few genes showing statistically significant alterations in their expression. However, a general trend towards inhibition of the ATRA-dependent effects, with particular reference to *Clusters-3*/*-5*/*-7*, is observed in *RA1sh-NB4* cells. This may be partially explained by incomplete silencing of RARα1 (see Figure 2A-2B).

We performed pathway enrichment analysis of the genes significantly regulated in *PMRsh-NB4* (comparisons c and g/d) and *RA2sh-NB4* cells (comparisons b and f/d) using annotated gene-collections (*Molecular Signatures* database) (Supplementary Table S2). In the 3 gene-collections considered, we found a significant overlap between the gene-sets enriched in *PMRsh-NB4* and *RA2sh-NB4* cells (Supplementary Figure S6A). Many of the genes regulated in *PMRsh-NB4* and *RA2sh-NB4* cells are direct PML-RAR targets [19] (Supplementary Figure S6B). The significant overlap between genes regulated by PML-RAR or RARα2 silencing and those regulated in NPM1-mutated blasts [38] (Supplementary Figure S7) may be of relevance for ATRA therapeutic action, since AMLs characterized by NPM1-mutations are deemed to be ATRA-sensitive [39–41]. Finally, enrichment in the genes of the GO “Immune-System-Process” (Supplementary Figure S8) may be linked to the neutrophil differentiation program triggered by PML-RAR and RARα2 knock-down. Indeed, emergency granulopoiesis is stimulated by inflammatory cytokines [42, 43], [44].

Finally, we evaluated the expression pattern of the genes contained in the “hematopoietic-cell-lineage” KEGG pathway (hsa04640) (Supplementary Figure S9), which is significantly enriched in genes regulated by PML-RAR (p<1E-6) and RARα2 (p<3.38E-4) silencing. As expected, the gene-regulation pattern observed following challenge with ATRA is consistent with myeloid differentiation. For instance, ATRA down-regulates CD135 (FLT3), a marker of hematopoietic stem-cells, while it up-regulates the neutrophil/monocyte marker, CD11b (ITGAM). Many ATRA-regulated mRNAs are also modulated by PML-RAR and RARα2 knock-down in the same direction.

### RARα2 interferes with the transcriptional activity of RARα1 and PML-RAR

**Figure 7:**
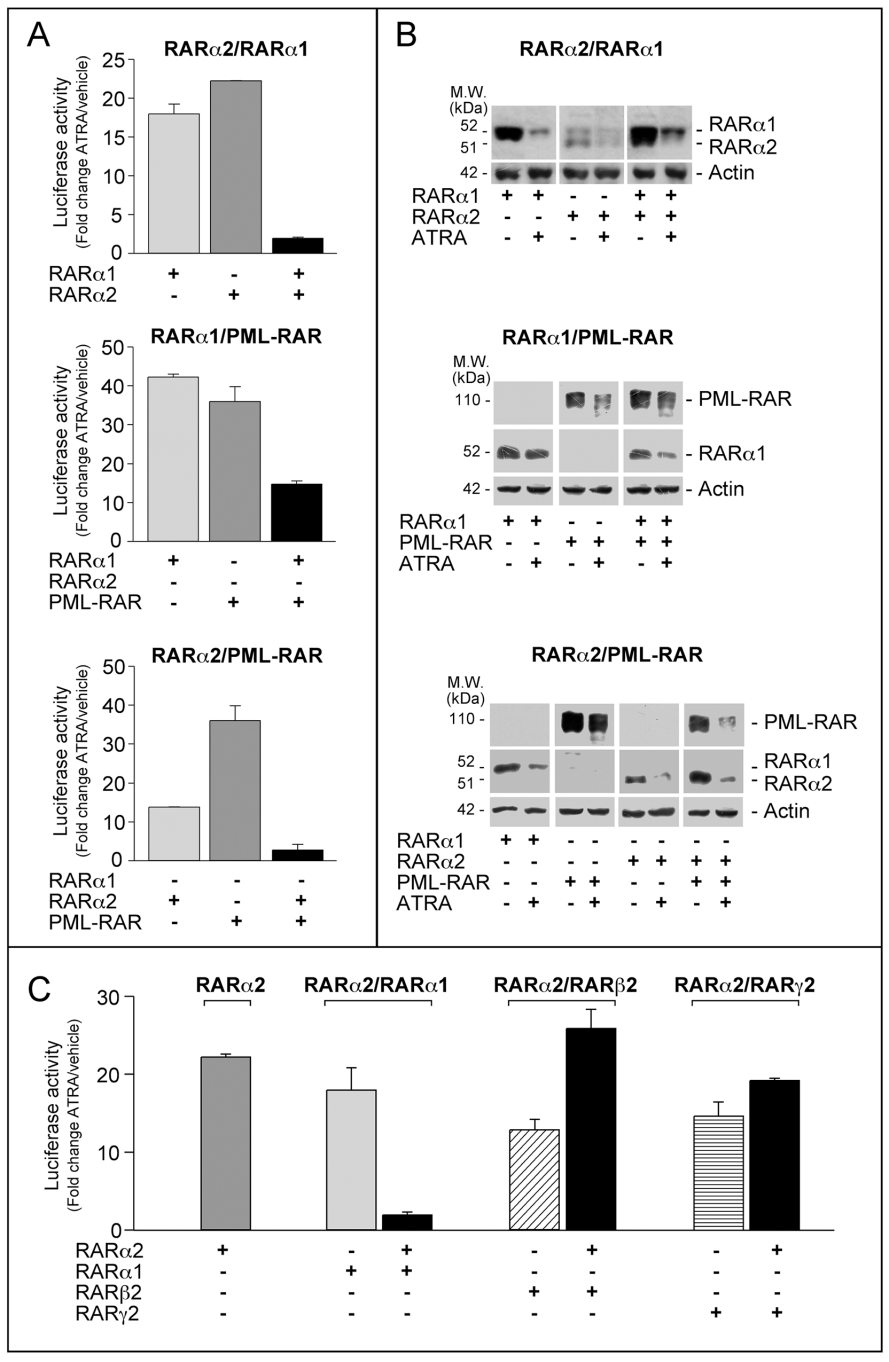
Interference between RARα2 and RARα1 or PML-RAR transcriptional activity **A**. *COS-7* cells were transfected with *pcDNA3-RARα2*, *pcDNA3-RARα1* and *pSG5-PML-RAR* plasmids alone or in combination and *β2RARE-Luc*. Sixteen hours following transfection, cells were treated with DMSO or ATRA (1 μM) for 24 hours. **B**. *COS-7* cell extracts were subjected to Western blot analysis with an anti-RARα antibody [RP alpha (F)]. The same amounts of extracts used for the determination of RARα1 and RARα2 were subjected to Western blot analysis to determine the loading control, β-actin. **C**. *COS-7* cells were transfected with *pcDNA3-RARα2*, *pcDNA3-RARα1*, *pSG5-RARβ2* and *pSG5-RARg2* plasmids alone or in combination and *β2RARE-Luc*. Sixteen hours following transfection, cells were treated with DMSO or ATRA (1 μM) for a further 24 hours. Luciferase activity is expressed as the ratio of ATRA/DMSO luciferase activity (fold-change). Each value is the mean±SD of two replicates.

To evaluate whether *RARα2* inhibitory action on myeloid differentiation involves direct effects on RARα1 and PML-RAR transcriptional activity, we used a co-transfection approach in *COS-7* cells, a popular model characterized by no expression of PML-RAR or RARα2 and very low expression of RARα1 [17, 23, 26]. We performed co-transfection studies with RARα2 and RARα1 or PML-RAR in *COS-7* cells transiently expressing the *β2RARE-tk-Luc* reporter. Separate transfection of RARα1, PML-RAR or RARα2 stimulates ATRA-dependent luciferase activity (Figure 7A). Simultaneous over-expression of RARα2 and RARα1 or PML-RAR reduces this stimulation, indicating cross-interference. Cross-interference is not due to effects on the amounts of RARα2, RARα1 or PML-RAR measured following separate and combined transfection in *COS-7* cells exposed to vehicle or ATRA (Figure 7B). Cross-interference is specific to RARα2 and RARα1 or PML-RAR, as indicated by the results obtained with RARβ2 or RARγ2. Indeed, co-transfection with RARα2 increases or leaves unaffected luciferase activity as compared to separate RARα2, RARβ2 or RARγ2 transfection (Figure 7C).

### RARα2 binds to RARα1 and PML-RAR directly: insights into the structural determinants of these interactions

**Figure 8:**
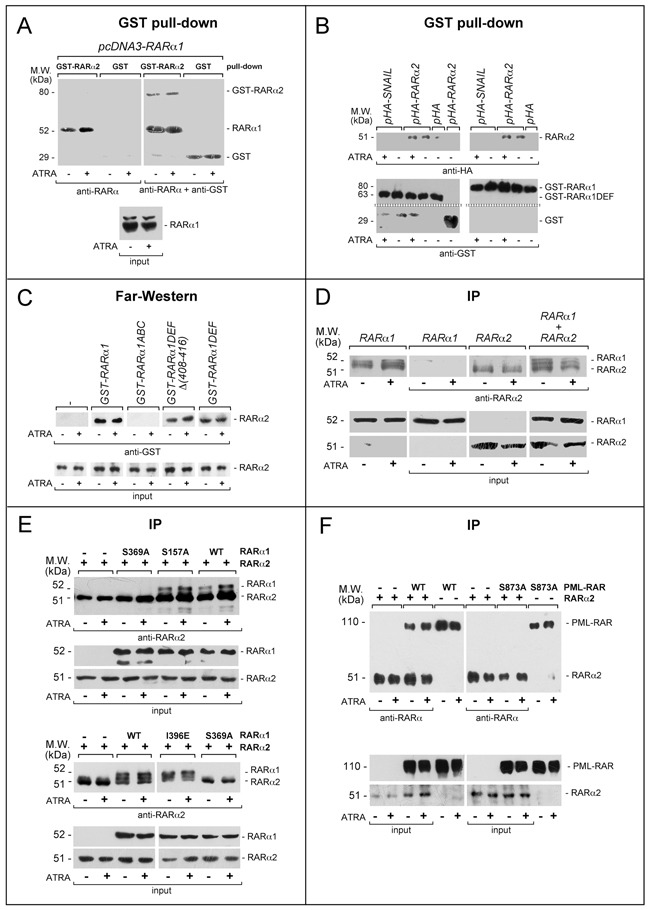
Functional and physical interactions between RARα2 and RARα1 or PML-RAR **A**. GST pull-down: the GST-tagged recombinant protein, GST-RARα2 and GST were used. The two recombinant proteins conjugated to Glutathione-Sepharose beads were incubated with extracts of *COS-7* cells transfected with *pcDNA3-RARα1* and treated with vehicle or ATRA (1 μM) for 4 hours. GST pull-down precipitates were blotted on nitro-cellulose filters, hybridized with an anti-RARα [RP alpha (F)] (left panel) and subsequently with an anti-GST antibody (right panel). The blot was not stripped between the two hybridizations. Input: cell extracts (10 μg of protein) representing 10% of the total amount of protein were subjected to Western blot analysis with the above anti-RARα antibody. **B**. GST pull-down: the GST-tagged recombinant proteins, *GST-RARα1* and *GST-RARα1DEF* were used. The two recombinant proteins conjugated to Glutathione-Sepharose beads were incubated with extracts of *COS-7* cells transfected with *pHA-RARα2* as well as the negative controls, *pHA-SNAIL* plasmid and *pcDNA3* plasmid containing the HA tag (*pHA*). Transfected cells were treated with vehicle or ATRA as in (A). As an internal control of the experiment, we performed a pull-down assay with the GST protein coupled to Glutathione-Sepharose beads on extracts of cells transfected with *pHA-RARα2*. GST pull-down precipitates were subjected to Western blot analysis with anti-HA (upper panels) and anti-GST antibodies (lower panels). **C**. Far-Western: *COS-7* cells were transfected with a *pcDNA3* plasmid containing a haemoagglutinin (HA) tagged RAR*a*2 cDNA (*pHA-RARα2*). Cell extracts were precipitated with agarose beads conjugated with an anti-HA monoclonal antibody. The immuno-precipitates were subjected to Far-Western analysis using the following GST-tagged RARα1 recombinant proteins: *GST-RARα1* = full-length RARα1; *GST-RARα1ABC = RARα1* ABC regions; *GST-RARα1DEF* = *RARα1* DEF regions; *GST-RARα1DEFD(408-416)* = *RARα1* DEF regions lacking the H12 helix. Input: cell extracts (10 μg of protein) representing 10% of the total amount of protein used for the immune-precipitations were subjected to Western blot analysis with an anti-HA antibody. Each line shows cropped lanes of the same gel, hence the results can be compared across the lanes, as they were obtained with the same exposure time. **D**. Immunoprecipitations (IP): *COS-7* cells were transfected with *pcDNA3-RARα1* and *pcDNA3-RARα2* alone or in combination. Sixteen hours following transfection, cells were treated with vehicle or ATRA (1 μM) for 4 hours. The indicated extracts were immuno-precipitated with anti-*RARα2* antibodies and subjected to Western blot analysis with a different anti-RARα antibody [RP alpha (F)]. The two leftmost lanes represent controls of RARα1 transfected cells directly submitted to Western blot analysis without immuno-precipitation. Equivalent amounts of protein extracts were used to immuno-precipitate RARα2, as indicated by the levels of RARα2 [Ab25alpha2(A2) antibody] and RARα1 [Ab10alpha1(A1)antibody]in the extracts (input). Each line shows cropped lanes of the same gel, hence the results can be compared across the lanes, as they were obtained with the same exposure time. **E**. Immuno-precipitation (IP): *COS-7* cells were co-transfected with wild-type (WT) RARα2 or WT RARα1 and RARα1 mutants and subjected to immune-precipitation and Western blot analysis as in (D). **F**. Immuno-precipitation (IP): *COS-7* cells were co-transfected with wild-type (WT) RARα2 or WT PML-RAR and derived mutant. The extracts of transfected cells were treated and subjected to co-immuno-precipitation studies as in (D and E). Lanes 5,6, 11 and 12 represent controls of PML-RAR and PML-RAR-S873A transfected cells directly submitted to Western blot analysis without immuno-precipitation.

To establish whether cross-interference involve interactions between RARα2, RARα1 and/or PML-RAR, we performed pull-down experiments in *COS-7* cells transfected with RARα1. Vehicle and ATRA-treated *COS-7* extracts were incubated with glutathione-S-transferase(GST)-tagged RARα2 (GST-RARα2) or control GST (Figure 8A). Upon WB of the GST-RARα2 pull-down fraction with an anti-RARα antibody, a band corresponding to transfected RARα1 is visible regardless of ATRA treatment. A similar band is not detected after GST pull-down. Superimposable results are observed if RARα1 is substituted by PML-RAR (data not show). Specular pull-down studies with *GST-RARα1* and *GST-RARα1DEF* (a *RARα1* recombinant product consisting of the entire D/E/F) on extracts of *COS-7* cells transfected with *pHA-RARα2*, *pHA-SNAIL* and *pHA* confirm and extend the results (Figure 8B and Supplementary Figure S10A). In fact, the data obtained following WB with anti-HA antibodies indicate that RARα2 is pulled-down by both *GST-RARα1* and *GST-RARα1DEF*. ATRA does not affect this interaction. Similar experiments conducted with *GST-RARα1ABC* do not result in RARα2 pull-down (data not shown). The specificity of the interaction between RARα2 and the D/E/F regions of RARα1 is supported by the results obtained in *COS-7* cells transfected with the negative *pHA-SNAIL* and the positive *pSG5-RXRα* controls (Supplementary Figure S10B).

Far-Western experiments were performed (Figure 8C) on extracts of *COS-7* cells transfected with hemoagglutinin(HA)-tagged RARα2 and treated with vehicle or ATRA. Immobilized HA-RARα2 immuno-precipitates (anti-HA antibodies) were challenged with GST-RARα1. Following incubation with GST-RARα1, but not GST, an anti-GST antibody highlights a band at the height of HA-RARα2. This demonstrates a direct interaction between HA-RARα2 and GST-RARα1, which is reproduced with the 2 GST-RARα1 derivatives consisting of the DEF regions containing (*GST-RARα1DEF*) or lacking the H12 helix [*GST-RARα1DEFD(408-416)*]. The GST-RARα1 protein consisting of the *ABC* regions (*GST-RARα1ABC*) does not interact with HA-RARα2. No quantitative difference in the RARα2/RARα1 interaction is observed in the absence/presence of ATRA.

To confirm the results obtained, we performed co-immuno-precipitation experiments in *COS-7* cells transfected with RARα2 alone or in combination with RARα1 using the specific anti-RARα2 antibodies. This was followed by WB with the distinct antibody [RPalpha(F)] detecting both RARα2 and RARα1. Co-precipitation of RARα1 (Figure 8D) is observed only in cells co-transfected with RARα2. The amounts of co-precipitated RARα1 are not influenced by ATRA treatment. RARα2 is immuno-precipitated only from cells transfected with the corresponding construct, confirming the specificity of the anti-RARα2 antibody. To get insights into the structural determinants of RARα2/RARα1 direct interaction, we performed similar co-immuno-precipitation experiments in *COS-7* cells co-transfected with RARα2 and selected RARα1 mutants of critical residues in the D/E/F regions (Figure 8E). Co-precipitation of RARα2 is observed in cells co-transfected with wild-type RARα1, the RARα1-S157A mutant (affecting a phosphorylation site at the C/D regions interface) and RARα1-I396E mutant (influencing the interactions with co-activators) [47]. In contrast, the RARα1-S369A mutant affecting the protein-kinase-A, mitogen-and-stress-activated-protein-kinase and p38-kinase phosphorylation site [23, 32, 48] does not interact with RARα2. The amounts of co-precipitated RARα1 and derived mutants are not significantly influenced by ATRA. Similar immuno-precipitation studies performed after co-transfection of RARα2, wild-type PML-RAR and the PML-RAR-S873A (corresponding to RARα1-S369A) confirm the above results. In fact, RARα2 co-immuno-precipitates only with wild-type PML-RAR (Figure 8F).

**Figure 9:**
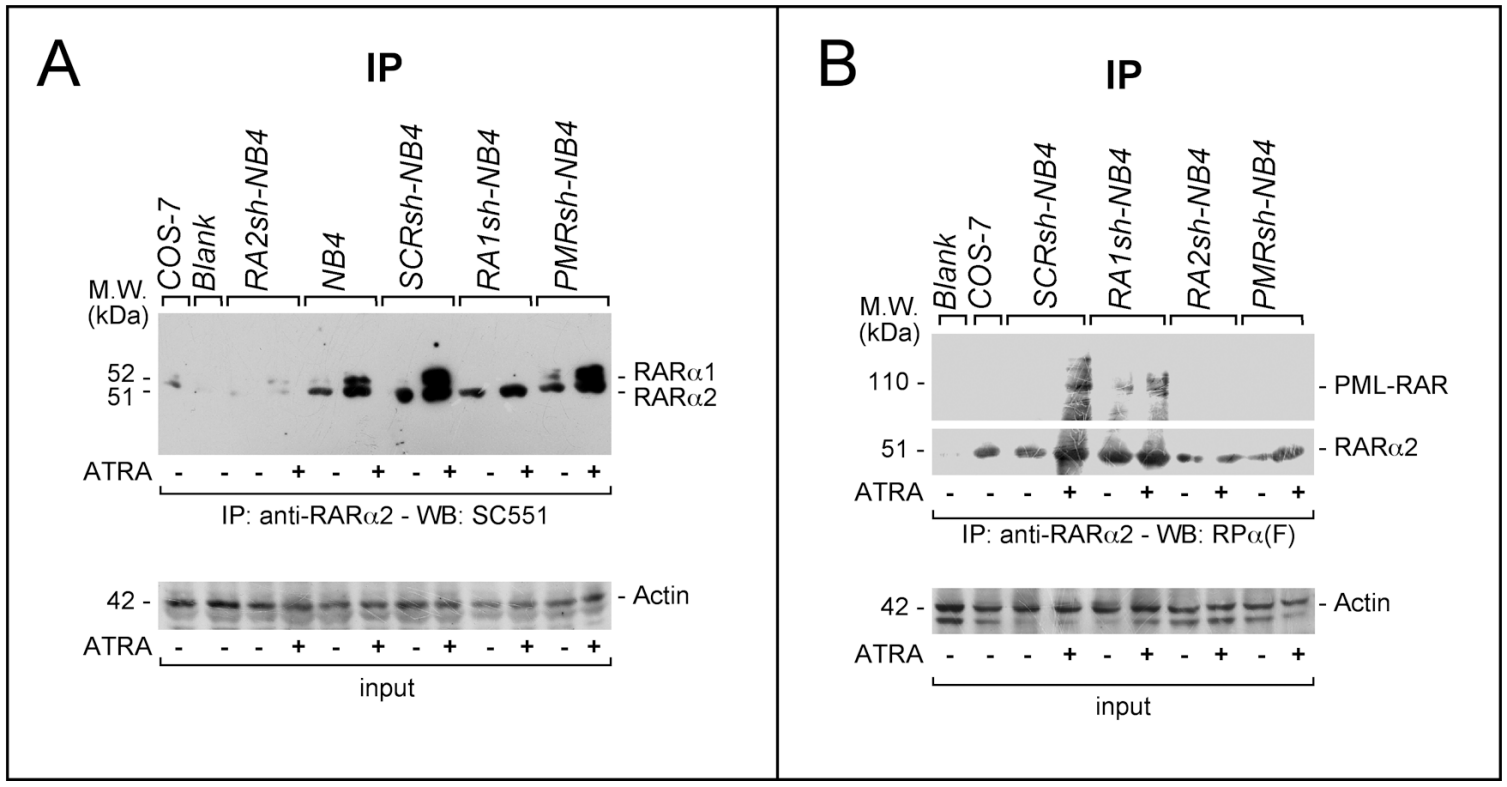
Physical interactions between RARα2 and RARα1 or PML-RAR in NB4 cells **A**. and **B**. Immuno-precipitation (IP) experiments: *SCRsh-NB4*, *RA1sh-NB4*, *PMRsh-NB4*, *RA2sh-NB4* and *NB4* parental cells were treated with vehicle or ATRA (1 μM) for 4 hours. Cell extracts were immuno-precipitated using an anti-RARα2 antibody [Ab25alpha2(A2)]. This was followed by Western blot analysis of the immunoprecipitates with two distinct antibodies, which, in our experimental conditions, detect RARα2 and RARα1 (SC551) (**A**) or RARα2, RARα1 and PML-RAR [RP alpha (F)] (**B**), respectively. Input: cell extracts (10 μg of protein) representing 10% of the total amount of protein used for the immune-precipitations were subjected to Western blot analysis with an anti-actin antibody.

To evaluate whether interactions between RARα2 and RARα1 or PML-RAR are also observed in the native APL context, we performed further immuno-precipitation studies in our *NB4* models. RARα2 is immuno-precipitated in extracts from parental, *SCRsh-NB4*, *RA1sh-NB4* and *PMRsh-NB4* but not in *RA2sh-NB4* cells. An extra 52 kDa band is detectable in the immuno-precipitates of untreated *NB4, SCRsh-NB4* and *PMRsh-NB4* only upon long exposures and its intensity is dramatically increased by ATRA (Figure 9A). The band corresponds to RARα1, as it is absent in *RA1sh-NB4* cells. To evaluate whether RARα2 is capable of interacting with PML-RAR, WB experiments on the anti-RARα2 immuno-precipitates were conducted with the antibody detecting PML-RAR, RARα2 and RARα1 (Figure 9B). In untreated *SCRsh-NB4* and *RA1sh-NB4* cells, PML-RAR and RARα2 co-immuno-precipitate. In the same cells treated with ATRA, the amounts of co-immuno-precipitated PML-RAR increase. The co-immuno-precipitated PML-RAR band is observed in neither untreated nor ATRA treated *RA2sh-NB4* and *PMRsh-NB4* cells.

In conclusion, our data demonstrate that RARα2 is capable of binding to RARα1 and PML-RAR and this binding may be at the basis of the observed functional interferences. The binding interface is located in the D/E/F region of RARα1 where the Ser-369 phosphorylation site plays a pivotal role in RARα2/RARα1 interaction. Binding of RARα2 to RARα1 and functional inhibition of the latter receptor may also partially explain the similarity in the effects afforded by RARα2 and PML-RAR silencing in *NB4* cells.

## DISCUSSION

It is believed that ATRA therapeutic action in APL involves degradation of PML-RAR [7, 49], which releases the suppressive effect exerted by the fusion protein on the product of the intact *RARA* allele, RARα [2, 3]. However, the existence of 3 RARα isoforms (RARα1, RARα2 and RARα4) adds complexity to the system. In the APL-derived *NB4* cellular model, RARα2 and PML-RAR are negative regulators of the granulocytic differentiation program and act *via* common transcriptional mechanisms. The similarities between RARα2 and PML-RAR activities are observed not only in *NB4* blasts under basal conditions, but also upon exposure to ATRA. Indeed, RARα2 and PML-RAR knock-down enhances ATRA-dependent induction of myeloid markers and the expression of direct retinoid target-genes. By converse, RARα1 knock-down does not alter the basal differentiation state and gene-expression pattern of *NB4* blasts, while it attenuates the ATRA-dependent induction of myeloid markers and the transcriptomic effects triggered by the retinoid. In *NB4* cells, the negative effects of RARα2 on ATRA differentiating activity are confirmed by over-expression approaches. As RARα2 is a negative prognostic factor in multiple myeloma [50, 51], the oncogenic action of RARα2 may extend to other hematological malignancies and solid tumors. With respect to this, we have preliminary evidence that over-expression of RARα2 in a retinoid-responsive breast-cancer cell line inhibits ATRA-simulated activity of a RARE-containing reporter and suppresses ATRA-dependent induction of the retinoid targets, SMAD3 and β-catenin [52, 53].

Our gene-expression studies provide insights into the molecular mechanisms underlying RARα2 and PML-RAR involvement in the process of granulocytic maturation induced by ATRA in APL blasts. In physiological conditions, the process is constitutively active during steady-state granulopoiesis, which is regulated by G-CSF, and GM-CSF [54, 55], and it is episodically stimulated during stress granulopoiesis, which is part of the innate immune response to infection/inflammation. Steady-state granulopoiesis requires the PU.1 and C-EBPα transcription factors [56–58], while stress granulopoiesis is stimulated by inflammatory cytokines like interferon [42, 43], IL-6, IL-3 and IL-1 [44]. ATRA seems to induce *NB4* cell differentiation *via* activation of both steady-state and stress granulopoiesis, since it induces PU.1, cEBPβ and IL-1. As for steady-state granulopoiesis, ATRA-dependent PU.1 induction is enhanced by PML-RAR and RARα2 knock-down, while it is blocked by RARα2 over-expression. By converse, cEBPα is down-regulated by ATRA and this is consistent with the absence of GM-CSF induction [59]. As for stress granulopiesis, PML-RAR and RARα2 knock-down up-regulates the basal expression and enhances ATRA dependent induction of numerous inflammatory genes with particular reference to those involved in the interferon pathway, including STAT1 (Supplementary Table S1). Constitutive and ATRA-induced c/EBPβ protein as well as IL-1β mRNA levels are enhanced by PML-RAR and RARα2 knock-down. Consistent with this, over-expression of RARα2 down-regulates both basal and ATRA-dependent expression of c/EBPβ.

Given the similarity of the effects induced by RARα2 and PML-RAR knock-down, the two receptors may act *via* common mediators or interact functionally. Functional studies confirm that RARα2, PML-RAR and RARα1 activate the same RARE-containing reporter in an ATRA-dependent fashion. Unexpectedly, however, RARα2 and PML-RAR interfere with RARα1 in terms of ligand-dependent transcriptional activity. This may explain the similarities between RARα2 and PML-RAR in terms of *NB4* differentiation and gene-expression profiles. The observed functional interferences support the concept that RARα1 is indeed a potential target of RARα2 and PML-RAR activity in APL cells. The significance of this is sustained by the data obtained on direct retinoid targets, like CD38 (Figure 3) CYP26A1 and RARβ (Supplementary Figure S11) in *NB4* cells. Indeed, time- and ATRA-dependent induction of CYP26A1 and RARβ mRNAs is blocked by RARα1 and enhanced by RARα2 or PML-RAR knock-down. Functional antagonism is at least partially explained by the ability of RARα2 and PML-RAR to physically interact with each other and RARα1. With the exception of the immuno-precipitation assays performed in *NB4* cells, our results demonstrate that direct binding is already observed in the absence of ATRA and it is not affected by the retinoid. However, in the *NB4* model, the levels of the RARα2/RARα1, PML-RAR/RARα1 and RARα2/PML-RAR complexes are increased by ATRA. The increase may be ascribed to RARα2 and PML-RAR induction by the retinoid (Figure 1). The interaction between RARα2 and RARα1 occurs also in PML-RAR-negative *HL-60* cells (Supplementary Figure S5B). Direct binding between the RARα2 and RARα1 or PML-RAR involves the *D*/*E*/*F* regions. At present the binding interface is incompletely defined, although our data indicate that binding is influenced by phosphorylation of RARα1 Ser-369 and the corresponding Ser-873 residue of PML-RAR. The observation is of particular interest for the therapeutic use of ATRA as it may suggest rational combination strategies aimed at enhancing the anti-leukemic potential of the retinoid. In fact, RARα1 S369 is a target phosphorylation site of protein-kinase-A, mitogen-and-stress-activated-protein-kinase and p38-kinase [23, 32, 48]. From a therapeutic prospective, the action of combinations between ATRA and inhibitors of the three kinases should be specifically evaluated in appropriate pre-clinical models. With respect to this, it is noticeable that a p38-kinase inhibitor has been shown to boost the differentiating activity of ATRA in APL cells [32]. Thus, it would be tempting to speculate that at least part of the effect exerted by the p38-kinase inhibitor is related to suppression of the RARα2/RARα1 or RARα2/PML-RAR interactions. In conclusion, we propose that the RARα2/RARα1 and RARα2/PML-RAR heterodimers may be transcriptionally inactive and may explain at least part of the observed functional antagonisms.

## MATERIALS AND METHODS

### Reagents and constructs

MG132, and ATRA were from Calbiochem and Sigma. Custom-designed short hairpin RNAs (shRNAs) were purchased from Ambion (Supplementary Figure S2). The 5′- and 3′-end of each hairpin RNA contain an EcoRI and a BamHI site to allow oriented cloning into the (pGreenPuro, System Biosciences, Palo Alto, CA) retroviral vector. The cDNA of the RARα2 coding region, which was amplified from *NB4* cells, was cloned into the *pCDNA3* plasmid (Invitrogen) and transferred to the *pCDH-CMV* lentivirus vector (System Biosciences), utilizing the EcoRI-NotI sites.

### Cells and infection/transfection procedures

*NB4* [60], *HL-60* [61] and *COS-7* cells were cultured as described [36, 62, 63]. We generated *NB4* cell populations silenced for PML-RAR and the RARα isoforms infecting cells with the *SCRsh*, *PMRsh*, *RA1sh* and *RA2sh* retroviral vectors according to standard protocols (System Biosciences). Following infection, *NB4* cells were selected in RPMI medium containing 10% bovine serum and puromycin (1.0 μg/ml) for at least 15 days. Only *NB4* cell populations characterized by >95% positivity to GFP following FACS analysis were considered. Subsequent passages of the cell populations were performed in complete RPMI medium containing puromycin (0.5 μg/ml). To isolate *NB4* populations over-expressing RARα2, cells were electroporated with *pCDH-CMV* lentiviral vectors (System Biosciences) containing RARα2 (*pRA2*) using Neon Transfection (Invitrogen, Life Technologies). *HL-60* cells were infected as above with the *RA2sh*, *SCRsh* or the void (*Void*) retroviral vector.

### Gene expression microarrays and real-time reverse-transcription-PCR

The *SCRsh-NB4*, *RA1sh-NB4*, *RA2sh-NB4* and *PMRsh-NB4* cell populations were treated with (DMSO) or ATRA (0.1 μM ) for 48 hours. Total RNA was reverse transcribed, labeled and hybridized to whole-genome gene expression microarrays (G4851B, Agilent, Santa Clara, CA) as already described [22]. Fluorescent signals were quantified with a laser scanner (Agilent). The microarray raw data were deposited in the Arrayexpress database (The accession No. E-MTAB-4713). Real-time reverse-transcription-PCR (RT-PCR) was performed with Taqman gene expression assays (C/EBPε, Hs00152928_m1; CYP26A1, Hs00175627_m1; 18S endogenous control, 4333762F; RARβ2, Hs00977143_m1; Applied Biosystems). The amplimers and Taqman probes used for the reverse-transcriptase RT-PCR assays of RARα-v1 to RARα-v4 were obtained from Life Technologies Italia (Monza, Italy) as detailed in Supplementary Methods.

### RARα1, RARα2 and PML-RAR transactivation

*COS-7* cells were transfected with RARα1, RARα2 and PML-RAR, alone or in combination in the presence of the RARE-containing β2RARE-luciferase reporter [64]. The normalization plasmid is a renilla luciferase construct (Promega) [63].

### FACS analysis, antibodies, immuno-precipitation and WB analyses

CD11b, CD11c and CD38 surface markers were determined with a Fluorescence Activated Cell Sorter (FACS, Becton and Dickinson) [26, 32]. Rabbit anti-RARα polyclonal antibodies [RPalpha(F)] and anti-RARα1 mouse monoclonal antibodies [Ab10a(A1)] were previously described [65]. Anti-RARα2 mouse monoclonal antibodies were raised against a synthetic peptide (amino-acids 1-29). Both Ab10a(A1) and Ab25a(A2) were purified on sulfoLink gel columns (Pierce Chemical) coupled to the immunizing peptide [66]. The other anti-RARα (SC551), anti-β-actin, cEBPβ, and anti-STAT-1 antibodies were from Santa-Cruz-Biotechnology. Anti-PU.1 and anti-paxillin antibodies were from Cell Signaling and Transduction Laboratories, respectively. WB analyses were performed as previously described [26] [32]. Immuno-precipitations were performed with antibodies immobilized on Protein-G-sepharose (Amersham). Agarose beads coupled to anti-HA antibodies were from Sigma (A2095).

### Far-western and GST pull-down assays

*COS-7* cells were transfected with a *pcDNA3* plasmid containing haemoagglutinin (HA)-tagged RAR*a*2 (*pHA-RARα2*). Extracts were precipitated with agarose-conjugated anti-HA monoclonal antibodies. The immuno-precipitates were subjected to Far-Western analysis using GST-tagged recombinant proteins [32]. For the pull-down experiments [32], we used the described *GST-RARα1*, *GST-RARα2* and derived recombinant proteins. *GST-RARα2* was obtained from *E. coli* cells transformed with an appropriate RARα2 cDNA construct cloned in the EcoRI-Not1 sites of *pGEX4T2*. The recombinant proteins conjugated to Glutathione-sepharose beads (Amersham) were incubated with extracts of *COS-7* cells transfected with *pHA-RARα2*, *pHA-SNAIL*, *pcDNA3-RARα1*, *pSG5-PML-RAR* or *pcDNA3* containing the HA-tag (*pHA*) and *pcDNA3* plasmids, for 4 hours. Pulled-down proteins were subjected to WB analysis using anti-HA, anti-RARα or anti-GST antibodies.

## SUPPLEMENTARY MATERIALS FIGURES AND TABLES






