# Supplementary material for: RARα2 and PML-RAR similarities in the control of basal and retinoic acid induced myeloid maturation of acute myeloid leukemia cells
Source: Oncotarget. 2016 Jul 13;8(23):37041–60. doi: 10.18632/oncotarget.10556 (PMC5514891; doi:10.18632/oncotarget.10556)
Supplement: Supplementary file 1 [file oncotarget-08-37041-s001.pdf]

# RARα2 and PML-RAR similarities in the control of basal and retinoic acid induced myeloid maturation of acute myeloid leukemia cells

## SUPPLEMENTARY METHODS

### TAQMAN assays

The amplimers and Taqman probes used for the reverse-transcriptase RT-PCR assays of the following transcripts were custom synthesized by Life Technologies Italia (Monza, Italy): RARα-v1 (NM_000964- forward primer = 5′-GGAATCCTGAATCGAGCTGAGA-3′, nucleotides 36-57; reverse primer = 5′-CAGTTCTGTGAG CTGGCACTTT-3′, nucleotides 236-215; Taqman probe = 5′- AGCAGCATCACAGGACAT-3′, nucleotides 112-129); RARα-v4 (NM_001145302-forward primer = 5′-GCACCA GCTTCCAGTTAGTGGAT-3′, nucleotides 725-747; reverse primer = 5′-CTTGTTTCGGTCGTTTCTCACA-3′, nucleotides 791-770; Taqman probe = 5′-CACACCAT CCCCAGCCA -3′, nucleotides 752-768). The inventoried Taqman assays for the indicated transcripts were purchased from Life Technologies Italia: RARα-v2 (Hs00940453_m1), RARα-v3 (Hs00940455_m1).

## SUPPLEMENTARY FIGURES AND TABLES

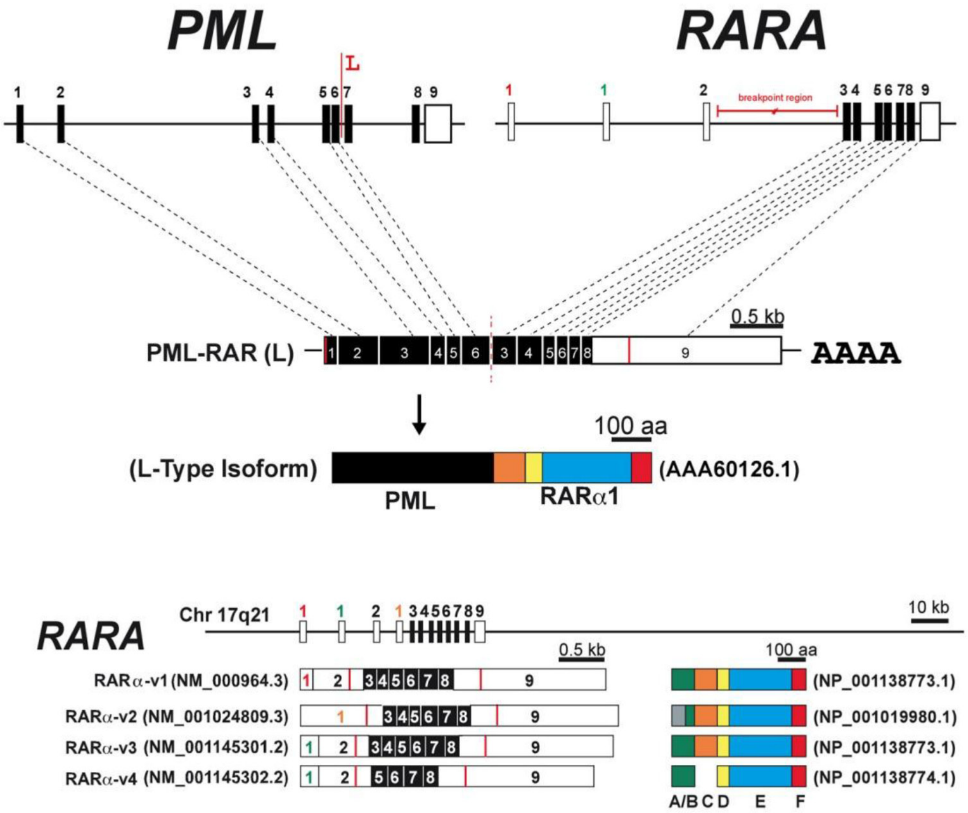


**Supplementary Figure S1: Structure of the *mRNAs* and proteins corresponding to the *PML-RAR* oncogenic chromosomal translocation and normal *RARA* gene.** The figure illustrates the structures of the indicated mRNAs and proteins along with the exon structure of the corresponding genes. The coding exons are represented by black boxes, while the 5′- and 3′-untranslated regions are indicated by white boxes. The accession numbers of the mRNAs and protein isoforms are indicated in parenthesis. The different regions of the RARα proteins are marked by colored boxes, while the black box of the PML-RAR protein indicates the portion encoded by the *PML* gene.

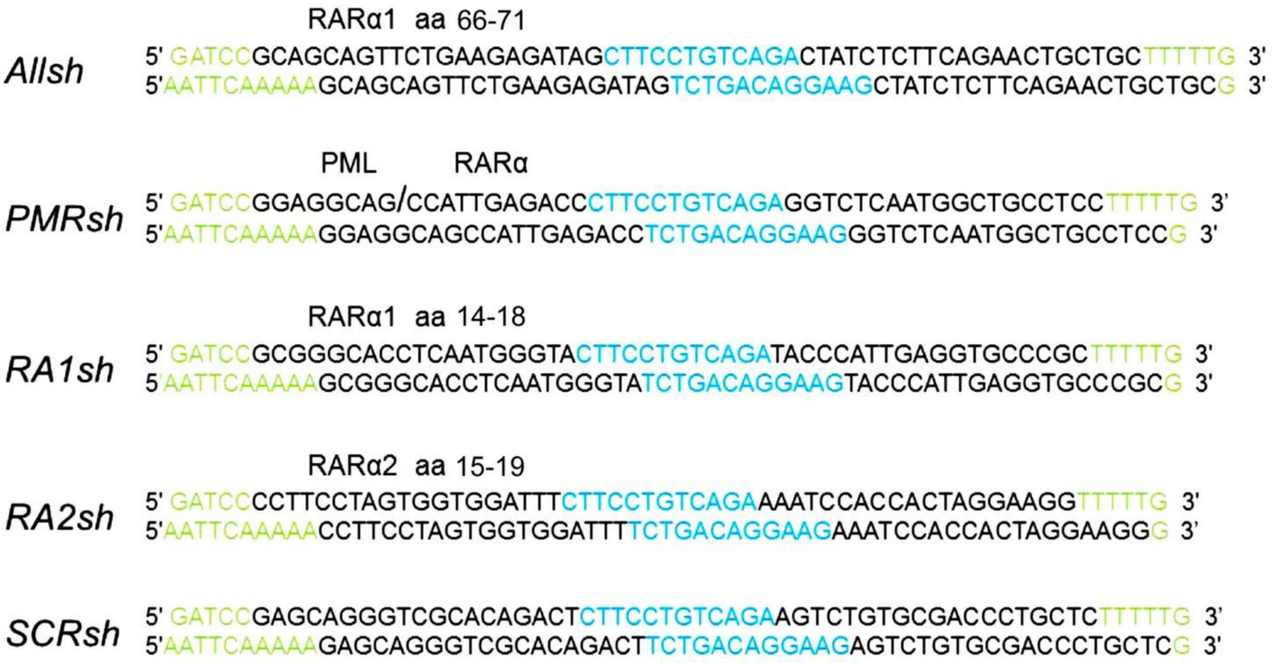


**Supplementary Figure S2: Structure of the double stranded *shRNAs* used in the study The figure illustrates the sequence of the indicated *shRNAs*.** The nucleotides marked in green correspond to the *EcoRI* and *BamHI* sites introduced for cloning purposes. The nucleotides marked in black correspond to the shRNA sequence, while those marked in light blue correspond to the joining loop. The position of the amino acids (aa) corresponding to the targeted sequences are indicated. *ALLsh* targets RARα1 amino acids 66-71 which are present also in the RARα2 and RARα4 proteins. *RA1sh* targets amino acids 14-18 which are specific of the RARα1 and RARα4 proteins. *RA2sh* targets amino acids 15-19 which are specific of RARα2. *PMRsh* targets the fusion point of the PML-RAR protein. *SCRsh*, acts as a negative control, since it consists of a scrambled sequence of *PMRsh*.

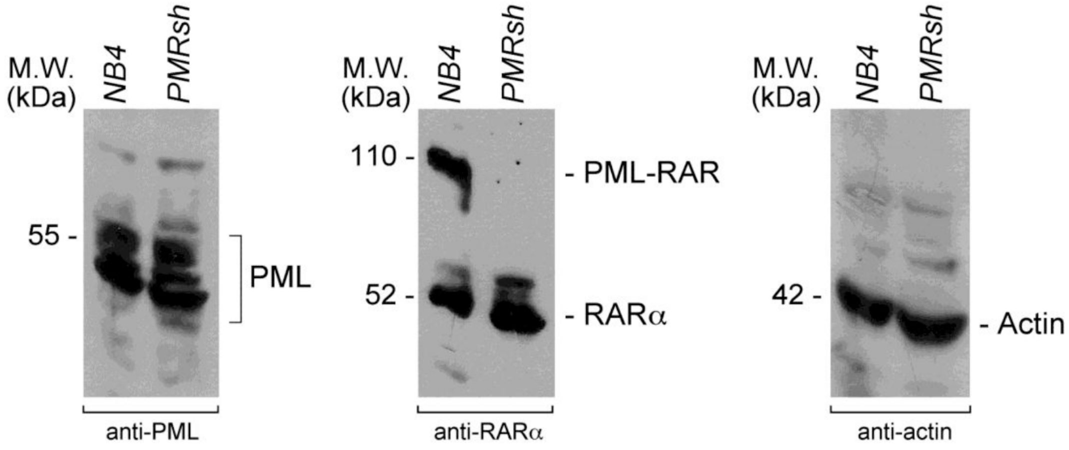


**Supplementary Figure S3: Lack of effects of PML-RAR knock-down on the expression of the native PML proteins in NB4 cells.** Extracts from parental *NB4* and *PMRsh-NB4* cells were subjected to Western blot analysis with anti-RARα and anti-PML antibodies. The signal intensity of the actin band obtained after subjecting the same amount of extract to Western blot is used as a loading control.

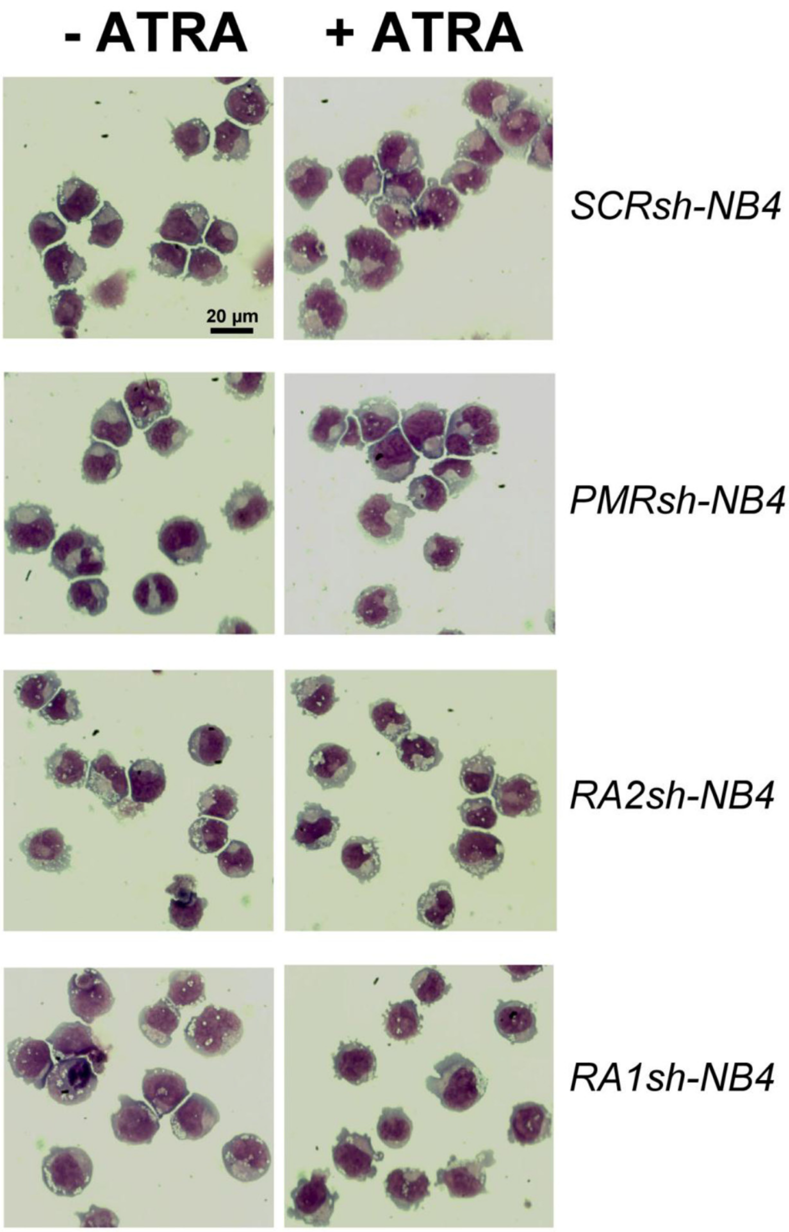


**Supplementary Figure S4: Morphology of in NB4 derived cells silenced for *PML-RAR, RARα1* and *RARα2*.** The indicated *NB4* cell populations stably infected with shRNAs targeting PML-RAR (*PMRsh-NB4*), RARα1 (*RA1sh-NB4*), RARα2 (*RA2sh-NB4*) or the control scramble shRNA (*SCRsh-NB4*) were grown in the presence of vehicle (DMSO) or ATRA (1 μM) for 72 hours. Cells were stained with May-Grunwald/Giemsa.

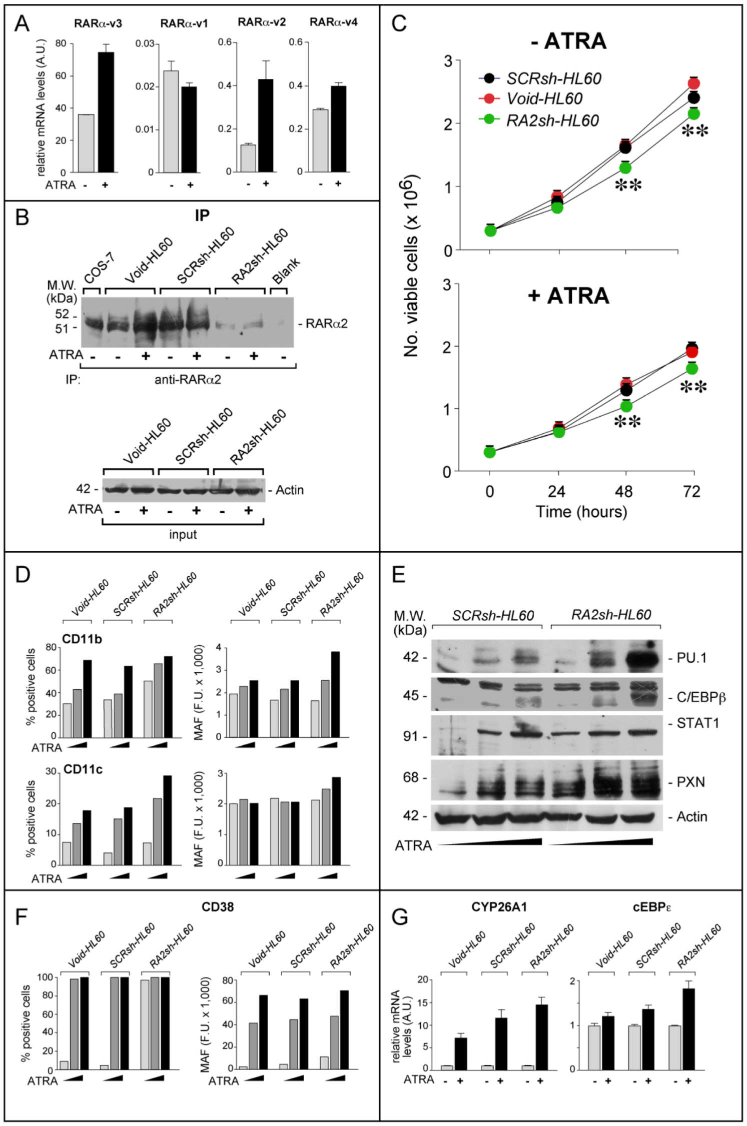

**Supplementary Figure S5: Effects of *RARα2* knock-down on the differentiation of *PML-RAR* negative HL-60 cells.** **A.** Logarithmically growing *HL-60* cells were treated with vehicle (DMSO) or ATRA (0.1 μM) for 48 hours. Total RNA was extracted and subjected to RT-PCR analysis using specific Taqman assays for the indicated RARα mRNAs. The results are expressed as mean±SD of 3 replicates. **B.** The indicated *HL-60* cell populations stably infected with shRNAs targeting RARα2 (*RA2sh-HL60*), control scramble shRNAs (*SCRsh-HL60*) or the void vector (*Void-HL60*) were treated with vehicle (DMSO) or ATRA (0.1 μM) for 48 hours. Cell extracts were immuno-precipitated with an anti-RARα2 antibody [Ab25alpha2(A2)] coupled to Protein G Sepharose beads. The immuno-precipitates were subjected to Western blot analysis with the anti-RARα antibody, [RP alpha (F)]. Equivalent amounts of protein extracts were used to immuno-precipitate RARα2, as indicated by the levels of actin in the extracts before addition of the anti-RARα2 antibody (input). **C.** Logarithmically growing cells were treated with vehicle (DMSO) or ATRA (0.1 μM) for the indicated amount of time. The number of viable cells determined after staining with trypan blue is indicated. Each point is the mean±S.D. of three replicate cultures. ** = Significantly different relative to the corresponding *Void-HL60* and *SCRsh-HL60* time points (p<0.01 after Student's t-test). **D.** and **F.** Cells were grown in the presence of vehicle (DMSO) or two increasing concentrations of ATRA (0.1 and 1.0 μM) for 72 hours and subjected to FACS analysis for the indicated markers. The column graphs show the percentage of CD11b-, CD11c- and CD38-positive cells and the MAF (mean-associated-fluorescence) values determined for each marker. **E.** The indicated cells were grown as in (D or F) and treated with vehicle or ATRA (0.1 and 1.0 μM) for 48 hours. Cell extracts were subjected to Western blot analysis for the indicated proteins. Actin is used as a loading control. The calculated molecular weight (M.W.) of each protein is indicated on the left. **F.** Cells were grown in the presence of vehicle (DMSO) or two increasing concentrations of ATRA (0.1 and 1.0 μM) for 72 hours and subjected to FACS analysis for the indicated markers. The column graphs show the percentage of CD11b-, CD11c- and CD38-positive cells and the MAF (mean-associated-fluorescence) values determined for each marker. **G.** Cells were treated with vehicle (DMSO) or ATRA (0.1 μM) for 48 hours. Total RNA was extracted and subjected to RT-PCR analysis using specific Taqman assays for the two indicated mRNAs. The results are expressed as the mean±SD of 3 replicates.

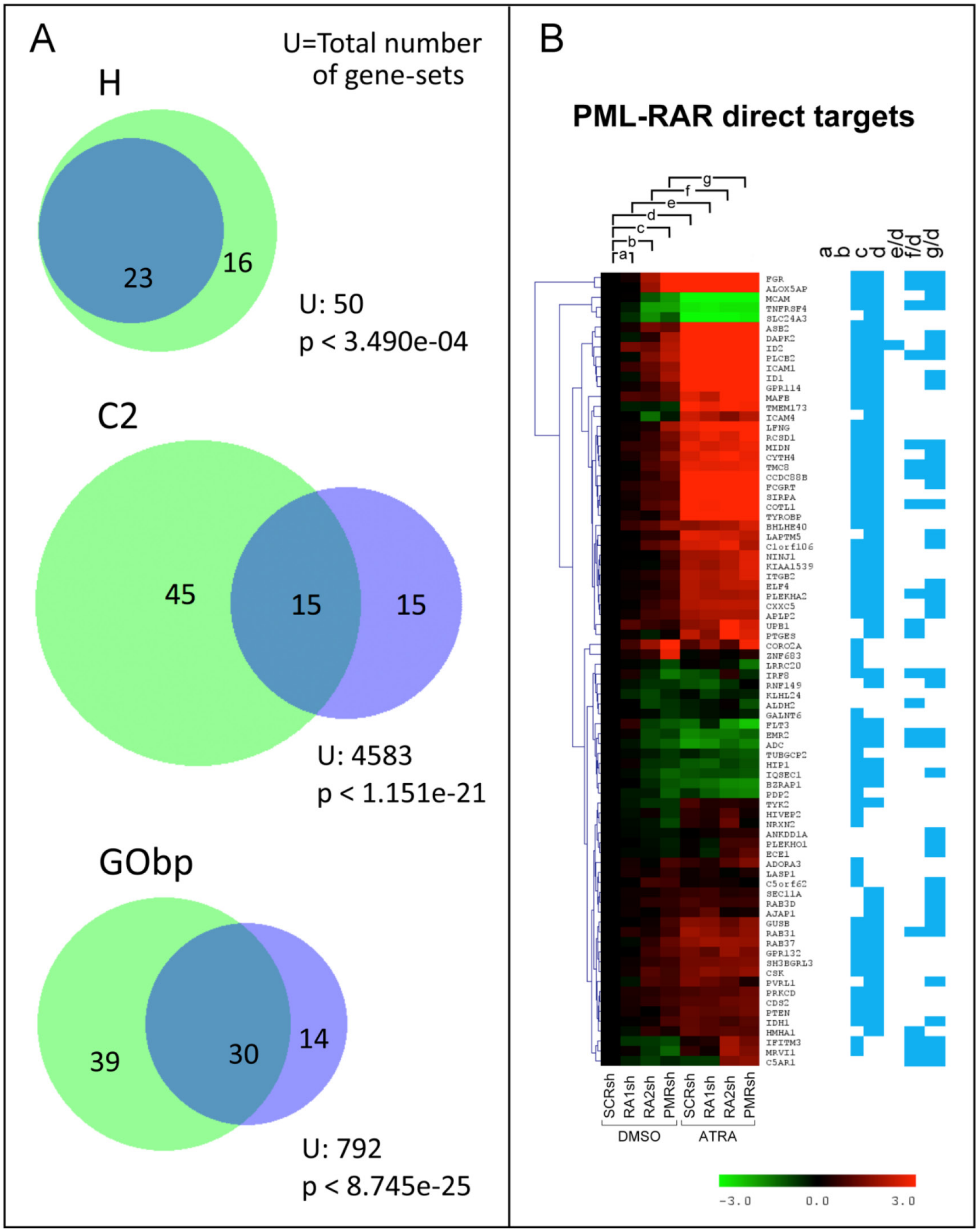


**Supplementary Figure S6: Pathway analysis of the genes regulated by *PML-RAR* or *RARα2* silencing.** We considered all the genes significantly regulated in *PMRsh-NB4* (comparisons c and g/d) and *RA2sh-NB4* (comparisons b and f/d) cells and performed pathway analysis using the annotated gene collections contained in the Molecular Signatures database: Hallmarks gene sets (H), which summarize and represent specific well-defined biological states or processes displaying coherent expression; Curated gene sets (C2), which are collected from various sources such as online pathway databases, publications in PubMed, and knowledge of domain experts; GO biological process (bp), which are derived from the Biological Process Ontology (http://www.geneontology.org). Due to the diverse numerosity of the collections, we used different p-values thresholds: H = 1.00E-02; C2 = 1.00E-18; GObp = 3.00E-04. **A.** Overlap between significantly over-represented gene sets in *PMRsh-NB4* and *RA2sh-NB4* cells are illustrated by the Venn diagrams (p-values refer to the hyper-geometric test). U = Total number of gene-sets present in each collection. The green and blue circles indicate the gene sets over-represented in *PMRsh-NB4* and *RA2sh-NB4* cells, respectively. **B.** Heatmap of the significantly regulated genes in the C2 collection gene-set: MARTENS_BOUND_BY_PML_RARA_FUSION (456 genes with promoters occupied by PML-RARA fusion protein in acute promyelocytic leukemia(APL) cells *NB4* and two APL primary blasts, based on Chip-seq data). This gene set is significantly enriched both in genes regulated by PML-RAR (p<1E-33) and RARα2 (p<1E-20) silencing. The statistical significance of the gene expression changes in the indicated comparisons is shown on the right by the blue lines.

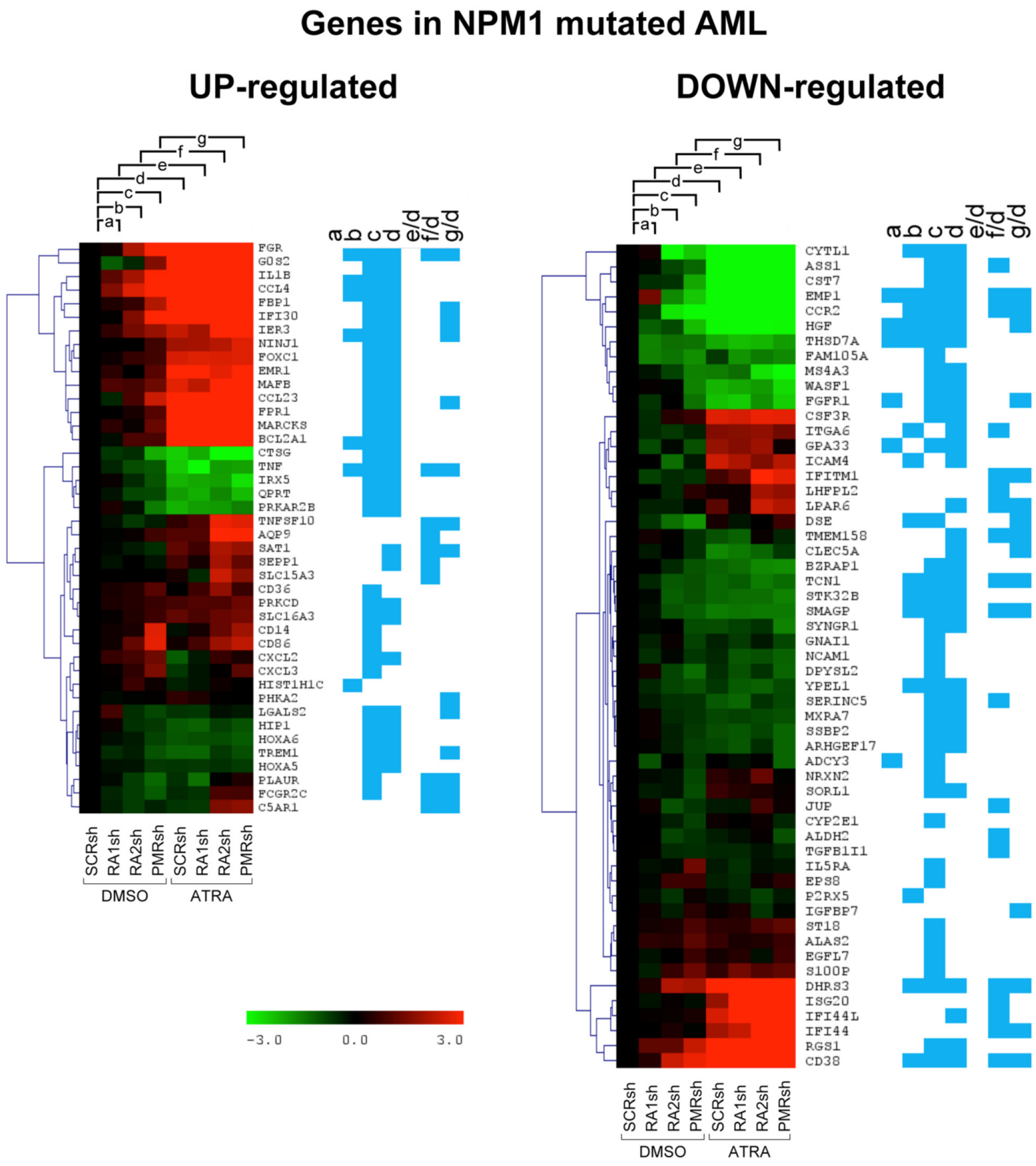


**Supplementary Figure S7: Pathway analysis of the genes regulated by *PML-RAR* or *RARα2* silencing – Enrichment in genes regulated in NPM1 mutated AML.** We considered all the genes significantly regulated in PMRsh-NB4 (comparisons c and g/d) and *RA2sh-NB4* (comparisons b and f/d) cells. We performed pathway analysis using the annotated gene collections contained in the Molecular Signatures database. The figure shows Heatmap of the significantly regulated genes belonging to the C2 collection gene sets VERHAAK_AML_WITH_NPM1_MUTATED_UP and _DN (183 and 246 genes respectively up- and down-regulated in acute myeloid leukemia (AML) patients with mutated NPM1). These gene sets are significantly enriched both in genes regulated by PML-RAR (UP = p<1E-23; DOWN = p<1E-24) and RARα2 (DOWN = p<1E-19) silencing. The statistical significance of the gene expression changes in the indicated comparisons is shown on the right by the blue lines.

## GO "Immune System Processes"

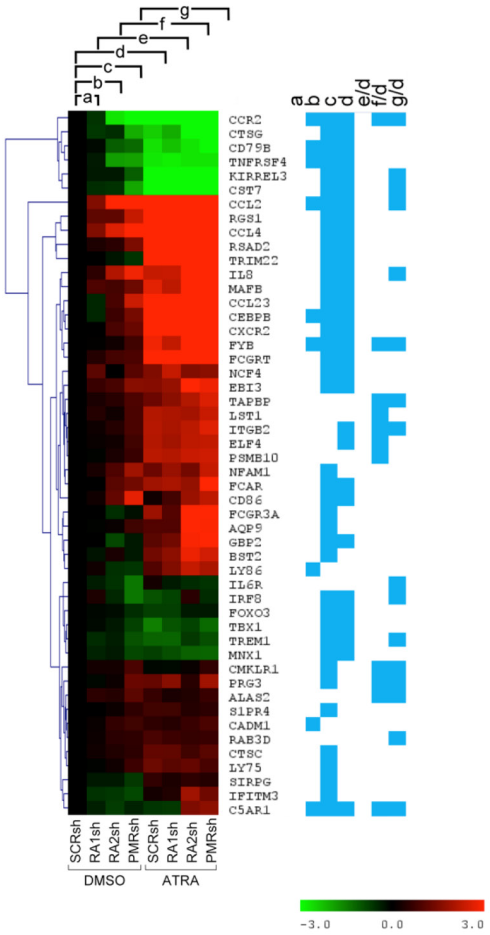


**Supplementary Figure S8: Pathway analysis of the genes regulated by *PML-RAR* or *RARα2* silencing– Enrichment in genes involved in immune system processes.** We considered all the genes significantly regulated in PMRsh-NB4 (comparisons c and g/d) and *RA2sh-NB4* (comparisons b and f/d) cells. We performed pathway analysis using the annotated gene collections contained in the Molecular Signatures database. The figure shows Heatmap of the significantly regulated genes belonging to the GO biological process IMMUNE_SYSTEM_PROCESS (332 genes annotated by the GO term GO:0002376 - Any process involved in the development or functioning of the immune system, an organismal system for calibrated responses to potential internal or invasive threats). This pathway is significantly enriched both in genes regulated by PML-RAR ($p<1E-19$) and RARα2 ($p<1E-7$) silencing. The statistical significance of the gene expression changes in the indicated comparisons is shown on the right by the blue lines.

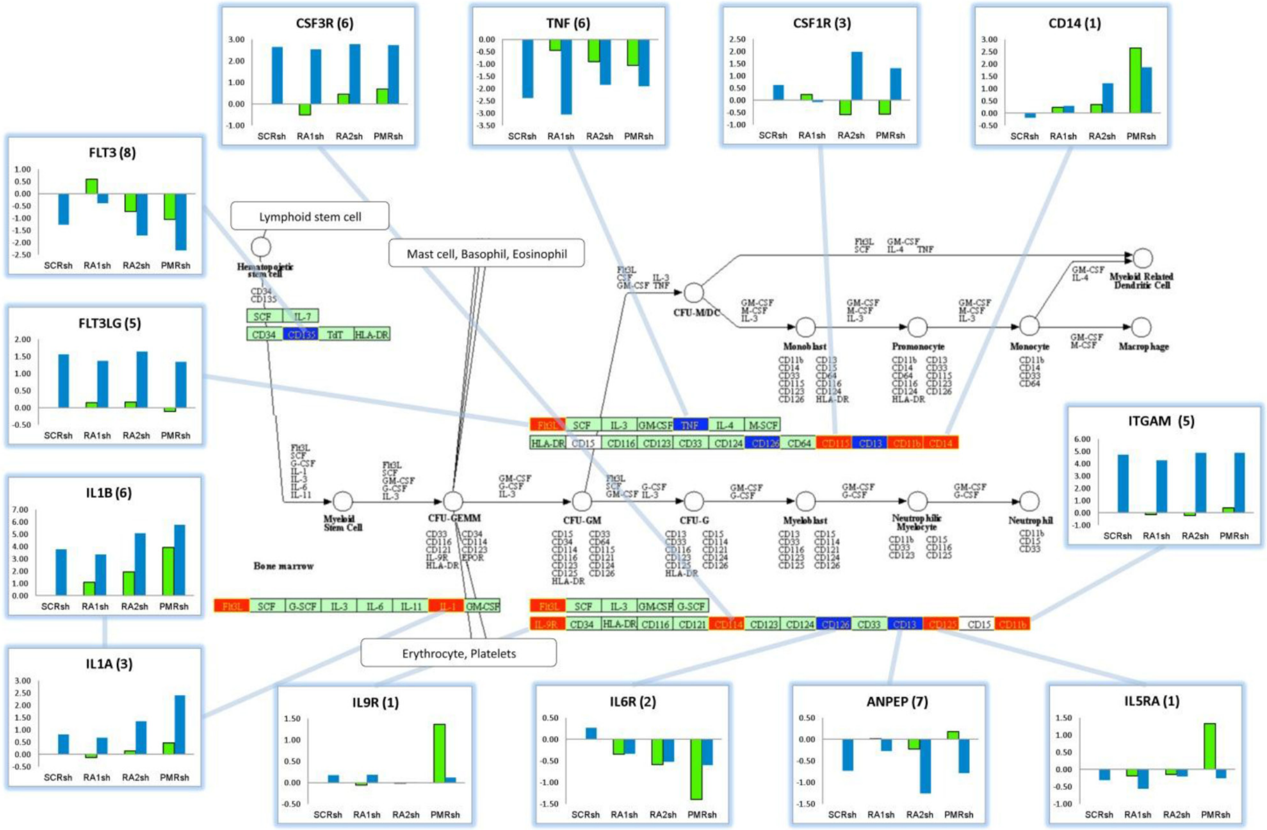


**Supplementary Figure S9: Pathway analysis of the genes regulated by *PML-RAR* or *RARα2* silencing – Enrichment in genes involved in hematopoietic differentiation.** We considered all the genes significantly regulated in *PMRsh-NB4* (comparisons c and g/d) and *RA2sh-NB4* (comparisons b and f/d) cells. We performed pathway analysis using the annotated gene collections contained in the Molecular Signatures database. The figure shows a section of the hematopoietic cell lineage KEGG pathway (hsa04640) relative to neutrophil and monocyte/macrophage differentiation. The expression pattern of the genes significantly regulated in our experimental conditions is shown in the bar graphs contained in the blue boxes. The green and blue columns represent the expression of each gene in basal conditions and after challenge with ATRA (0.1 µM) for 48 hours. For each experimental group, data are reported as the $\log_2$ of the ratio vs basal expression in SCRsh-NB4 cells (SCRsh_DMSO). The number in parenthesis indicates the cluster the gene belongs to.

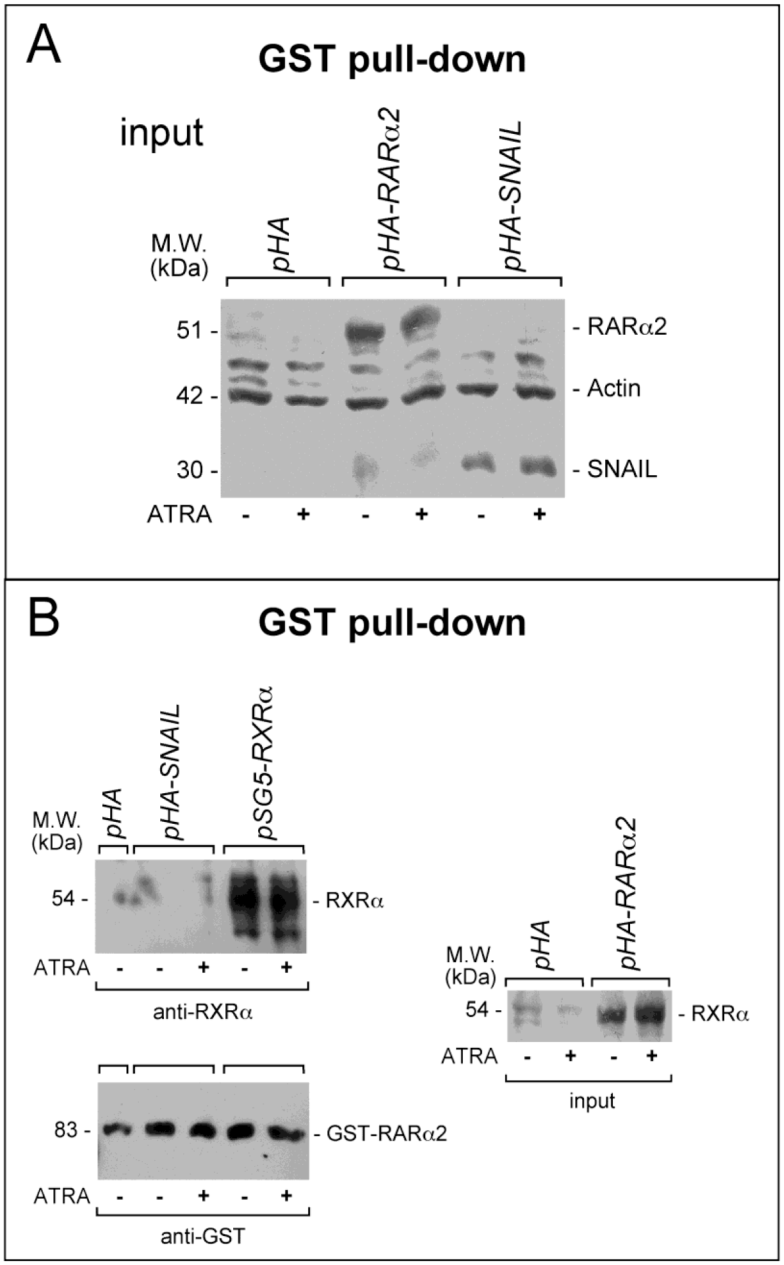


**Supplementary Figure S10: GST-pull-down experiments A.** The figure illustrates the input proteins of the GST pull-down experiment of Fig. 10B. *COS-7* cells were transfected with *pHA-RARα2*, *pHA-SNAIL* and a negative control *pcDNA3* plasmid containing the HA tag (*pHA*). After transfection, cells were treated with ATRA (1 µM) for 4 hours. Cell extracts (10 µg of protein) representing 10% of the total amount of protein used for the GST pull-down experiment were subjected to Western blot analysis with anti-HA and anti-actin antibodies. **B.** The recombinant GST-RARα2 protein conjugated to Glutathione-Sepharose beads was incubated with extracts of *COS-7* cells transfected with *pSG5-RXRα*, *pHA-SNAIL* and *pHA* plasmids. After transfection, cells were treated with ATRA (1 µM) for 4 hours. GST pull-down precipitates were subjected to Western blot analysis with anti-RXRα and anti-GST antibodies (upper and lower left panels). Cell extracts (10 µg of protein) representing 10% of the total amount of protein used for the GST pull-down experiment were subjected to Western blot analysis with anti-RXRα antibodies (input, right panel).

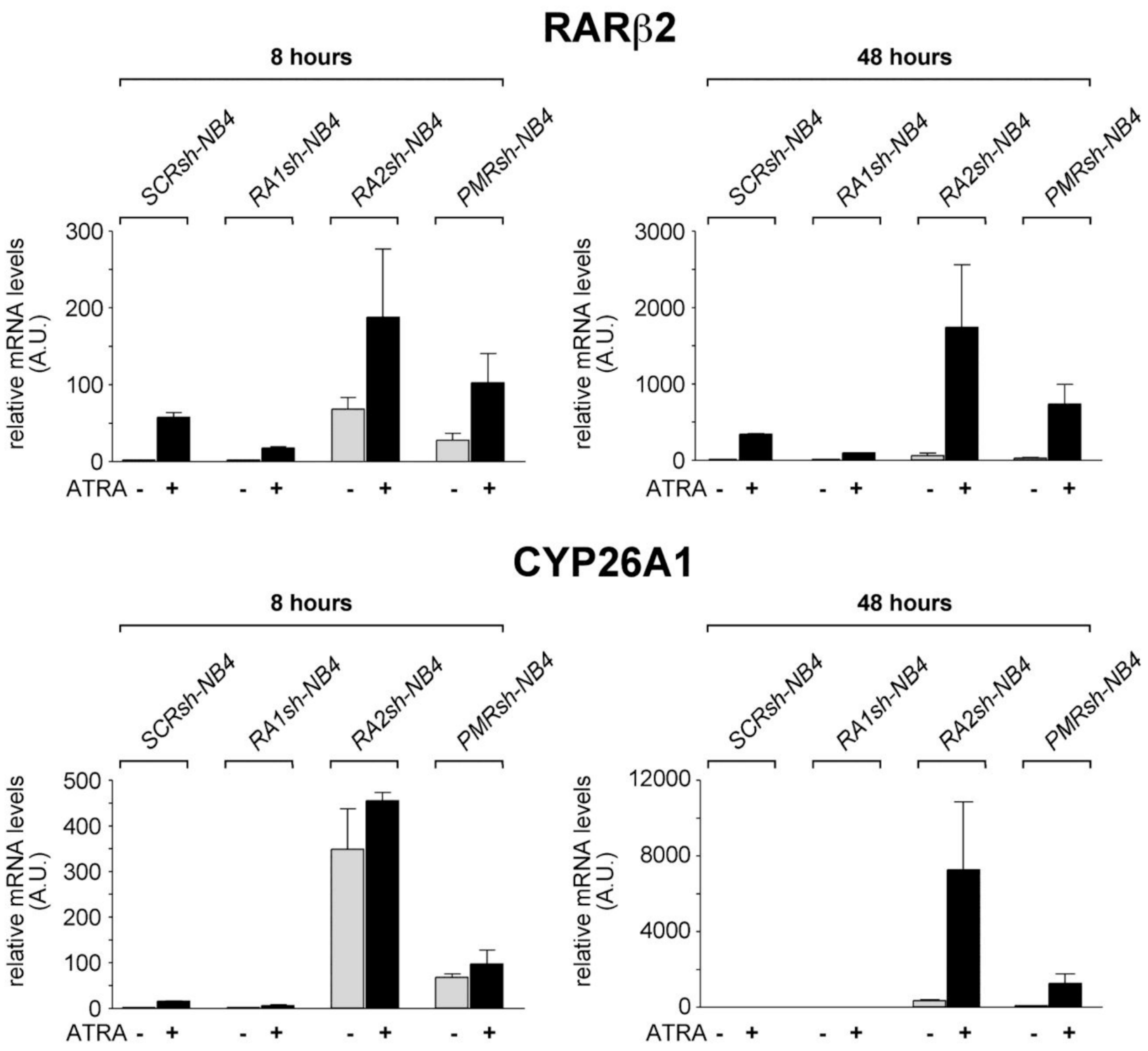


**Supplementary Figure S11: Time-dependent expression of *RARβ2* and *CYP26A1* mRNAs in NB4-derived cell populations silenced for RARα1, RARα2 and PML-RAR.** The indicated cell populations were treated with ATRA (0.1 μM) for the indicated amount of time. Total RNA was extracted and subjected to RT-PCR analysis for the determination of RARβ2 and CYP26A1. The following Taqman assays were used: RARβ2 = Hs00977143_m1; CYP26A1 Hs00175627_m1.

**Supplementary Table S1: Whole-genome gene expression profiles of *SCRsh-NB4, PMRsh-NB4, RA2sh-NB4* and *RA1sh-NB4* cells growing under standard conditions or following exposure to ATRA.** *SCRsh-NB4*, *PMRsh-NB4*, *RA2sh-NB4* and *RA1sh-NB4* cells were treated with vehicle (DMSO) or ATRA (0.1 µM) for 48 hours. Whole-genome gene expression experiments were performed with Agilent microarrays (G4851B). The table reports a list of the differentially expressed genes and the contrasts considered in the analysis (see Fig. 4 and Fig. 5 of the manuscript). The genes were grouped into eight clusters, indicated in the last column, according to their pattern of regulation.

**See Supplementary File 1**

**Supplementary Table S2: Gene set enrichment analysis in *PMRsh-NB4* and *RA2sh-NB4* cells.** The table reports significant overlaps between the genes regulated by silencing of PML-RAR and the gene setscollected in the Molecular Signatures Database (MSigDB, http://software.broadinstitute.org/gsea). Significance calculations were performed with the webtool provided by the Broad Institute (http://software.broadinstitute.org/gsea/msigdb/annotate.jsp). Due to the different numerosity of the collections, we used different p-values thresholds: H = 1.00E-02; C2 = 1.00E-18; GObp = 3.00E-04. For the significant overlaps we also report the results observed for RARα2 silencing. Green boxes indicate the sets for which we provide detailed information on the expression of the single genes (see Fig. EV1 Appendix Figs. S4 and S5). Orange boxes mark other functionally relevant gene sets.

**See Supplementary File 2**
